# What are the needs in oral antitumor therapy? An analysis of patients’ and practitioners’ preferences

**DOI:** 10.3389/fonc.2024.1388087

**Published:** 2024-06-27

**Authors:** Anna Hester, Franziska Henze, Anna Marie Debes, Charlotte Leonie Schubert, Alexander Koenig, Nadia Harbeck, Rachel Wuerstlein

**Affiliations:** Department of Obstetrics and Gynecology, Breast Center and Comprehensive Cancer Center (CCC) Munich, University Hospital, LMU Munich, Munich, Germany

**Keywords:** CDK4/6 inhibitor, oral therapy, patient education, patient preference, e-health, metastatic breast cancer, continuos regimen, 21/7 regimen

## Abstract

**Background:**

Since the European approval of CDK4/6 inhibitors in 2016, the treatment of patients with hormone-receptor-positive, HER2-negative metastatic breast cancer has changed significantly. Compared with chemotherapy, endocrine-based therapy has different treatment regimens and is associated with new side effects. Oral therapy aims for optimal drug efficacy and long treatment times while maintaining maximum independence and quality of life resulting in the conservation of medical staff resources.

**Methods:**

A monocentric analysis of therapy preferences of practitioners (25 nurses and physicians) and patients (11 on endocrine monotherapy, 17 on endocrine-based therapy, and 14 on intravenous chemotherapy) was performed using specific questionnaires. Preferences were assessed using a four-point Likert scale or bidirectional response options.

**Results:**

All patients were highly supportive of oral therapy (mean agreement score on the Likert scale 1.3, *p* < 0.001 *vs*. all other options) and a consultation interval of 4 weeks (2.0, *p* = 0.015 *vs*. 3 weeks). Practitioners also preferred oral therapy (1.4) and visits every 4 weeks (1.6). In general, patients on oral therapies reported higher compatibility of their therapy with daily life than patients on chemotherapy (1.6 and 1.7 *vs*. 2.6, *p* = 0.006). Outpatient oncology is the main source of information for all patients, mainly in case of side effects (2.0) and open questions (1.8). Regarding oral antitumor therapy regimens, patients do not show a significant preference for a specific regimen, while practitioners prefer a continuous regimen (1.6) over a 21/7 regimen (21 days on and 7 days off therapy, 2.5). Patients are likely to accept mild side effects (e.g., neutropenia, diarrhea, polyneuropathy, fatigue) and would still adhere to their initial choice of regimen (continuous or 21/7). Only when side effects occur with a severity of CTCAE grade 3 do patients prefer the regimen in which the side effects occur for a shorter period of time.

**Conclusion:**

Patients and practitioners prefer oral antitumor therapy—both continuous and 21/7 regimens—over other application forms. Patient education and proper therapy management, supported by additional tools, contribute to the specific management of side effects and high adherence. This allows quality of life to be maintained during long-term therapy with CDK4/6 inhibitors in patients with metastatic breast cancer.

## 1 Introduction

Breast cancer is the most common cancer in women worldwide with a lifetime risk of approximately 10% for women in Western countries. 4%–10% of all breast cancer patients present with primary metastatic disease, and approximately 20%–40% of the remaining patients develop metastases during the course of their disease (1). For clinical purposes and treatment decisions, breast cancer is biologically classified according to the presence or absence of expression of the hormone receptors (HRs) for estrogen and progesterone and a possible amplification of the human epidermal growth factor receptor 2 (HER2). HR-expressing (+) HER2 not amplificated (−) breast cancer is the most common subtype. Although it is often considered less aggressive than the other subtypes, recurrent disease or metastases can still occur a long time after primary diagnosis (2). The most common sites of metastasis in HR+ HER2− breast cancer are the bone, lung, liver, brain, and skin (3). With the development of new therapeutic options and the individualization of treatment regimens in recent decades, the therapy of primary and metastatic breast cancer has improved significantly. Treatment options for metastatic HR+ HER2− breast cancer have classically included oral or intramuscular endocrine therapies and oral or intravenous chemotherapies. However, due to recent therapeutic improvements, HR+ HER2− breast cancer can also be treated with oral, targeted therapies, such as the revolutionary cyclin-dependent kinase (CDK) 4/6 inhibitors (palbociclib, ribociclib, abemaciclib) (4). In addition, mammalian target of rapamycin (mTOR) inhibitors (everolimus), phosphatidylinositol 3-kinase (PI3K) inhibitors (alpelisib), and poly(ADP-ribose) polymerase (PARP) inhibitors (olaparib, talazoparib) are used in oral targeted therapy (5–8).

As of 2017, the standard first-line therapy regimen for patients with metastatic HR+ HER2− breast cancer is oral endocrine-based therapy with a classical endocrine therapy (aromatase inhibitor or fulvestrant) in combination with a CDK4/6 inhibitor and—in case of bone metastases—a bone-modifying drug (bisphosphonate or denosumab) (2). Chemotherapy, on the other hand, is only indicated in cases of visceral crisis (9, 10). Today, three different CDK4/6 inhibitors—palbociclib, ribociclib, and abemaciclib—are available for metastatic breast cancer.

All phase III trials of CDK4/6 inhibitors showed a significant increase in median progression-free survival (PFS) with CDK4/6 inhibitor therapy compared with placebo, but not all showed an increase in overall survival (OS) (4). Palbociclib (Ibrance^®^) became the first CDK4/6 inhibitor to be approved in Europe on 9 November 2016, based on data from the PALOMA trials: in the PALOMA-2 trial, postmenopausal patients with primary metastatic breast cancer received a combination of palbociclib and letrozole. PFS in the palbociclib/letrozole group increased significantly to 24.8 months compared with 14.5 months in the placebo/letrozole group (11). However, no statistically significant improvement in OS was observed after long-term follow-up (12). Ribociclib was evaluated in the MONALEESA study program: patients in the MONALEESA-2 trial benefited from endocrine-based therapy with ribociclib and letrozole with a PFS of 25.3 months compared with placebo/letrozole with a PFS of 16.0 months. OS was also significantly prolonged, with 63.9 months in the ribociclib arm compared with 51.4 months in the placebo arm (13, 14). Ribociclib (Kisqali^®^) was approved in Europe on 22 August 2017. The third CDK4/6 inhibitor, abemaciclib, also showed a significant and comparable improvement in PFS in combination with letrozole or fulvestrant in the MONARCH trials (15, 16). The final analysis of the MONARCH3 trial showed an increase in OS of 13.1 months compared with the control arm, which did not reach statistical significance (17). Abemaciclib was approved in Europe on 27 September 2018, under the trade name Verzenios^®^.

The various CDK4/6 inhibitors differ in their dosages, regimens, and potential side effects (**Table 1**). Palbociclib (standard dose: 125 mg) and ribociclib (standard dose: 600 mg) must be taken once daily for 21 consecutive days, followed by a 7-day rest before starting a new cycle (so-called “21/7 regimen”). Abemaciclib, on the other hand, is taken twice daily (at the standard dose of 150 mg) without a break (so-called “continuous regimen”). If side effects occur, the daily dose of all three CDK4/6 inhibitors can be reduced in two steps (see **Table 1**) according to the prescribing information (18–20).

**Table 1:** Dosing, treatment regimen, and most common side effects (≥ 30%, all grades) under therapy with palbociclib (*n* = 768), ribociclib (*n* = 1,065), and abemaciclib (*n* = 768) in combination with endocrine therapy with letrozole or fulvestrant according to the prescribing information.

	Palbociclib		Ribociclib		Abemaciclib
**Dosing**	125 mg, 100 mg, 75 mg	**Dosing**	600 mg, 400 mg, 200 mg	**Dosing**	150 mg, 100 mg, 50 mg
**Treatment regimen**	21/7 regimen, 1×/day	**Treatment regimen**	21/7 regimen, 1×/day	**Treatment regimen**	Continuous regimen, 2×/day
AE	% of all patients	AE	% of all patients	AE	% of all patients
Neutropenia	82.1	Neutropenia	74.3	Diarrhea	84.6
Infections	49.2	Nausea	51.5	Neutropenia	45.1
Leucopenia	48.6	Infections	50.3	Infections	43.6
Fatigue	41.5	Fatigue	36.5	Nausea	43.5
Nausea	36.0	Diarrhea	35.0	Fatigue	40.5
Stomatitis	30.3	Alopecia	33.2	Anemia	30.1
		Leucopenia	32.9		

Adapted after (18–20).

AE, adverse event.

The most common severe side effects of palbociclib [Common Terminology Criteria for Adverse Events (CTCAE) grade 3 or higher] as described in the prescribing information are neutropenia, leukopenia, and fatigue. In randomized clinical trials, 38.4% of patients receiving palbociclib required dose reductions or therapy adjustments due to adverse reactions, regardless of the endocrine combination partner (19). The most common grade 3 or grade 4 adverse events with ribociclib are neutropenia, leukopenia, abnormal liver function tests, QT time prolongation, nausea and vomiting, infections, and fatigue. Dose reductions due to adverse events were required in 37.3% of patients treated with ribociclib in phase III clinical trials, while 7% had to discontinue treatment permanently (20). Patients treated with abemaciclib mainly reported severe (CTCAE grade 3 or higher) side effects such as diarrhea, neutropenia, infections, leukopenia, abnormal liver function tests, nausea and vomiting, and fatigue. In contrast to the other two CDK4/6 inhibitors, diarrhea was significantly more common with abemaciclib, which may be pathophysiologically explained by the higher selectivity for CDK4 compared with CDK6 (18).

Regarding side effects, the prescribing information provides detailed instructions on therapy management including the required monitoring intervals. For palbociclib, a complete blood count is required before starting therapy, at the beginning of each cycle, on day 15 of the first two cycles, and as clinically indicated (19). For ribociclib, electrocardiograms (ECGs) and electrolytes should be monitored prior to initiation of treatment. ECGs should be repeated on approximately day 14 of the first cycle, at the beginning of the second cycle, and as clinically indicated. Electrolytes should be monitored at the beginning of each cycle for six cycles and as clinically indicated. Liver function tests (LFTs) and a complete blood count must be performed before starting treatment, every 2 weeks for the first two cycles, at the beginning of each of the subsequent four cycles, and as clinically indicated (20). For abemaciclib, complete blood counts and LFTs should be monitored prior to initiation of therapy, every 2 weeks for the first 2 months, monthly for the next 2 months, and as clinically indicated. Patients should be instructed to initiate antidiarrheal therapy, increase oral fluid intake, and notify their healthcare provider at the first sign of loose stools (18).

The fundamental change in the treatment of HR+ HER2− metastatic breast cancer from intravenous to oral tumor therapy and the long treatment periods with the new oral therapies pose new challenges for both practitioners and patients.

Since oral antitumor therapy with CDK4/6 inhibitors is taken independently by the patients in their home environment, extensive information and education of both patients and their families and caregivers is necessary prior to initiation of therapy (21): individual schedules—e.g., when to take the drug regularly or when to stop in case of a 21/7 regimen, or specifying the individual dose—should be discussed with both patients and their families. Important drug-specific features, such as interactions with over-the-counter medication, dietary supplements, and foods, also play an important role in targeted therapy and must be emphasized in discussions with patients. This information should be updated throughout the course of therapy. Detailed information about possible side effects of oral therapy should be explained to the patients and their families. To ensure adherence and safety, patients need a clear plan and detailed information about the regular check intervals (as described above) according to the prescribing information, and they need to know when to involve relatives or doctors, when to take additional medication, or even when to stop therapy in case of side effects. In addition to detailed pretreatment education, patients need to be followed closely during the treatment. They need regular and scheduled face-to-face visits to monitor adherence and discuss therapy details in person, but they also need emergency contact numbers and information options available at all times of the day (21).

New logistical challenges for some patients include the compatibility and flexibility of therapy-related appointments when returning to work. Early and seamless vacation planning can also be a challenge: if patients are planning to go on vacation, the most common side effects and their management in an emergency should be reviewed in detail, all relevant information and contact details should be available in writing, and prescriptions for therapy and concomitant medications should be available for the duration of the vacation. In addition to the new demands on the patient in terms of self-responsibility, the treatment team is also faced with new challenges. Often, logistical and personnel restructuring is necessary to ensure continuous and high-quality patient care. Patients can generally be accompanied by either nurses or physicians. In addition, several digital tools have recently been introduced to monitor and manage therapy. Whereas in the past patients had to use calendars or written notes to document their therapy and possible side effects, digital tools are now available for this purpose (22). Similarly, various websites and apps support patients with detailed information and coaching modules for therapy accompaniment To our knowledge, there has been no detailed analysis of patients’ preferences for personal therapy accompaniment and management.

The aim of the study was to evaluate preferences regarding oral antitumor therapy and therapy accompaniment in a real-world setting. The study was conducted at the Breast Cancer of the LMU University Hospital in Munich, Germany. Both patients and practitioners (nurses and physicians) were interviewed to assess what they expect from oral antitumor therapy, what kind of therapy accompaniment they prefer, and which therapy regimen is the most preferred in which clinical situation. We even assigned two different treatment schedules with equal therapy efficacy (an on/off schedule and a continuous schedule) with side effects of increasing severity and analyzed whether these side effects influence the preference for a specific treatment schedule. To our knowledge, no such analysis has been performed so far. Our results may help in counseling patients when choosing a CDK4/6 inhibitor for therapy—as they have all shown comparable efficacy, other factors need to be taken into account when making therapy decisions. In addition, the results on therapy accompaniment preferences may help practitioners caring for these patients in their daily clinical practice.

## 2 Materials and methods

### 2.1 Survey

Patients with metastatic breast cancer treated with either endocrine monotherapy, endocrine-based therapy, or chemotherapy at the Breast Center of the Department of Gynecology and Obstetrics of the LMU University Hospital in Munich, Germany, and practitioners/healthcare professionals (physicians and nurses) working at the same center were eligible for this project. The voluntary survey was conducted between December 2020 and March 2021 by distributing questionnaires to the study population, after the project was approved by the LMU Ethics Committee (ethical approval number 21-0848). The return date for the questionnaires was May 2021. The respective questionnaires were developed specifically for this study and are available in the **Supplementary Files**. The original questionnaires were in German and were translated for submission.

The “endocrine monotherapy” group included all patients receiving either letrozole, anastrozole, exemestane, or fulvestrant (in combination with a gonadotropin-releasing hormone analog if premenopausal). The “endocrine-based therapy” group included patients who received a CDK4/6 inhibitor (either palbociclib, ribociclib, or abemaciclib) in combination with one of the endocrine therapies above. Patients in the “chemotherapy” group received a classic intravenous or oral chemotherapy, such as paclitaxel, epirubicin/cyclophosphamide, or capecitabine.

The patient questionnaires covered the following areas in the first section: demographics, information about the disease, information about current therapy, and questions about therapy accompaniment and treatment regimen preferences. Multiple response options were available for the accompaniment and treatment regimen preference questions. Questions had predefined response options or were open-ended. Questions on treatment preferences were scored on a Likert scale from 1 to 4 (1 = strongly agree, 2 = somewhat agree, 3 = somewhat disagree, 4 = strongly disagree).

The second section was designed to assess patients’ preferences for different CDK4/6 inhibitor regimens (21/7 regimen *vs*. continuous regimen). However, instead of specifically asking patients which CDK4/6 inhibitor and which intake regimen they preferred, two hypothetical but identically acting oral drugs should be compared by the patients. We did this to avoid confounding by possibly already known drug names. Patients were clearly told that both drugs were expected to have the same oncologic efficacy, regardless of their intake regimen. These hypothetical drugs therefore stood as substitutes for CDK4/6 inhibitors. One of these hypothetical drugs was to be taken continuously, the other in a 21/7 regimen. In 21 different questions, each drug was supposed to have a specific side effect. These side effects were neutropenia (and subsequent increased risk of infection), diarrhea, fatigue, and paresthesia in the fingers and toes (polyneuropathy). Each side effect could hypothetically occur with a severity of CTCAE grade 1, 2, or 3. Each side effect could occur for 2 days in the continuous scheme and for 7 days in the 21/7 regimen in a 28-day period (referred to in the analysis as “shorter duration of side effect in the continuous regimen”) or vice versa (referred to in the analysis as “shorter duration of side effect in the 21/7 regimen”). In each question, patients were asked to decide if they would rather take drug A or drug B under the given condition. In this manuscript, the choice of a drug is shown in case of a grade 1 or grade 3 side effect (grade 2 is omitted for clarity).

The practitioner questionnaires included questions about demographics, the scope of practice at the breast center, and professional experience in oncology. Practitioners were also asked about their preferences for different treatment regimens, using a Likert scale with the same coding as above. As part of this survey, the practitioners were asked additional questions, the results of which are not presented in this publication. Therefore, the attached questionnaire for practitioners has been shortened to the questions relevant to this publication.

### 2.2 Statistics

Questionnaires were analyzed using Microsoft Excel and SPSS software. For patient and practitioner characteristics, data from questions with predefined responses were summarized, and median and range were presented when applicable (e.g., age, years of therapy, years of experience). Data from the Likert-scale questions in the questionnaire (results in 3.2.1, 3.2.2, and 3.3) were analyzed as follows: the mean and standard deviation of the value on the Likert scale were calculated for each question, both for the overall cohorts of patients and practitioners and for the predefined subgroups (endocrine monotherapy, endocrine-based therapy, and chemotherapy and physicians and nurses). These results are described in this manuscript as “mean approval score.” Differences between the subgroups regarding the mean approval score for each question were analyzed using the Kruskal–Wallis test (for more than two subgroups) and the Mann–Whitney *U* test (for two subgroups). Differences in the overall cohort on multiple variables (e.g., weekly *vs*. 3 weekly *vs*. 4 weekly) were analyzed using ANOVA.

Data from the questions in the questionnaire with only two response options (analysis of the second section regarding treatment regimen: 21/7 or continuous, results in 3.2.3) were analyzed for differences between the subgroups using chi-squared tests. *p*-values ≤0.05 were considered statistically significant.

## 3 Results

### 3.1 Characteristics of the patients and practitioners

The basic characteristics of the patients interviewed are shown in **Table 2**. Eleven patients on endocrine monotherapy were interviewed. They were generally the oldest patients (median age 71 years), more often already retired (81.8%), and had the lowest metastatic burden with mainly bone metastases. The 17 patients on endocrine-based therapy were generally younger (median age 66 years), more often still working (23.5% retired), and also had predominantly bone metastases. The 14 patients receiving chemotherapy were the youngest (median age 54.6 years) and most likely to be working part-time or full-time (only 21.4% were retired). They had the highest metastatic load, including major organ metastases (brain, liver).

**Table 2:** Basic characteristics of the patients surveyed in this study.

	Endocrine monotherapy (*n* = 11)	Endocrine-based therapy (*n* = 17)	Chemotherapy (*n* = 14)
**Age, years (median, range)**	71 (44, 84)	62.3 (35, 80)	54.6 (32, 71)
**Time between primary diagnosis and metastases, years (median, range)**	9.9 (0, 17)	6.8 (0, 22)	3.7 (0, 25)
**Localization of metastases, percentage (*n*)**	81.8% (9) bone 18.2% (2) lung/pleural 18.2% (2) lymph nodes 9.1% (1) skin	82.4% (14) bone 29.4% (5) lung/pleural 17.6% (3) liver 5.9% (1) lymph nodes	64.3% (9) bone 28.6% (4) lungs/pleural 42.9% (6) liver 14.3% (2) peritoneum 14.3% (2) lymph nodes 14.3% (2) brain 7.1% (1) pericardium

The basic characteristics of the surveyed practitioners (both physicians and nurses) are shown in **Table 3**. Most were experienced practitioners, working in the field of oncology for at least 5 years.

**Table 3:** Basic characteristics of the practitioners surveyed in this study.

	*n*	%			
Number of practitioners	25	100	Age, years (median, range)	Years working in oncology	
**Physicians**	11	44.0		n	
Specialized oncologist	5	20.0	43.6 (33, 57)	3	>10 years
				2	5–10 years
Resident	6	24.0	31.0 (28, 36)	2	5–10 years
				4	< 10 years
**Nurses**	14	56.0	46.9 (31, 64)		
Nurse specialized in oncology	2	8.0		5	>10 years
Breast care nurse	2	8.0		5	5–10 years
General nurse	5	20.0		4	< 5 years
Physician’s assistant	5	20.0			

We describe the preferences of the patients and practitioners interviewed in this study as their mean agreement score on a Likert scale to different options as explained in the *Methods* section (1 = strongly agree, 2 = partially agree, 3 = partially disagree, 4 = strongly disagree).

### 3.2 Patient preferences

#### 3.2.1 Patient preferences regarding therapy accompaniment and management

##### 3.2.1.1 Therapy management tools

**Figure 1:**
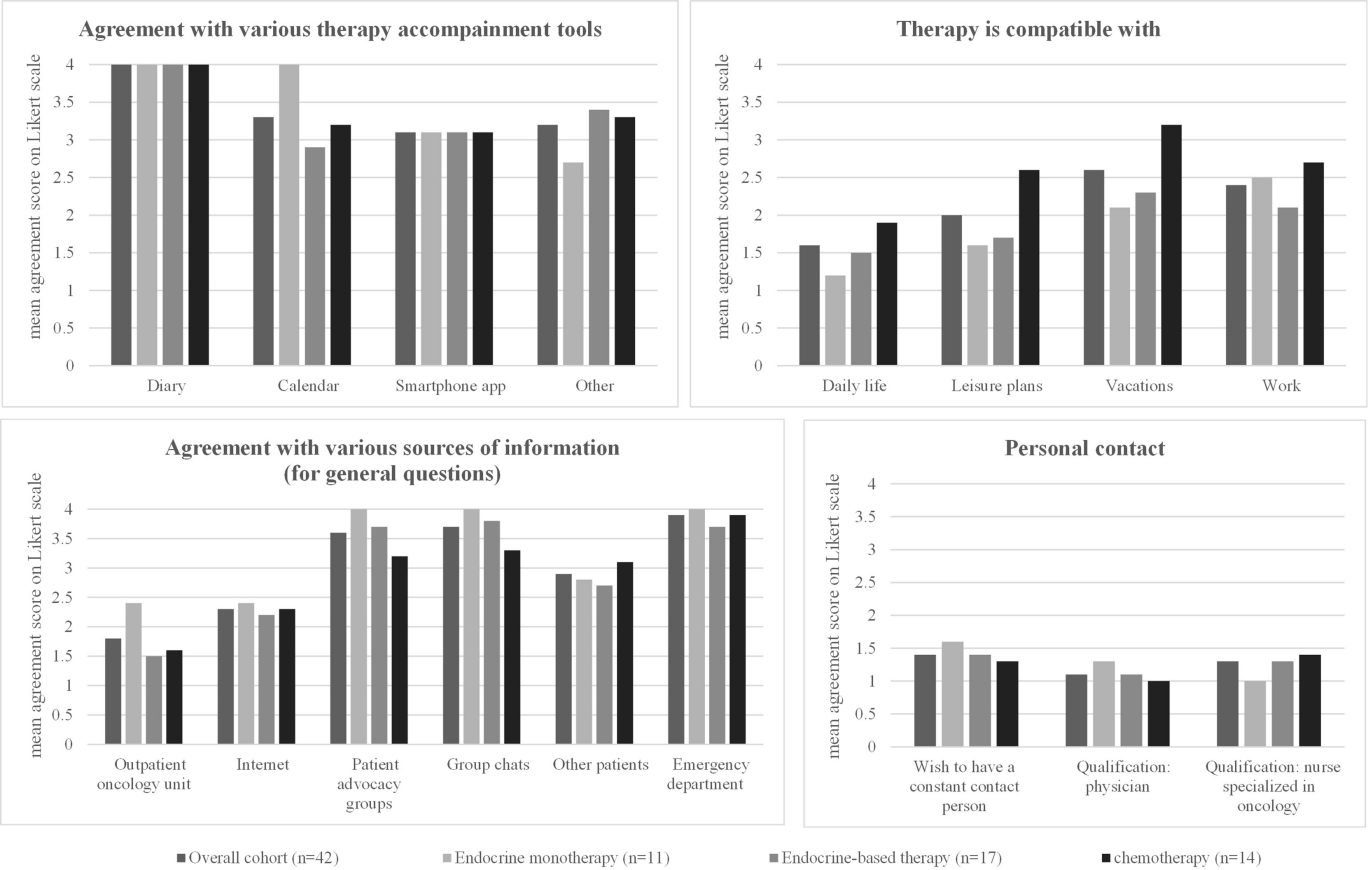
Patient preferences regarding side effects. Left: The number of pills (mean) already taken and accepted to reduce side effects is shown. Right: For each option, the mean rating on a four-point Likert scale (1 = strongly agree, 2 = partially agree, 3 = partially disagree, 4 = strongly disagree) in the overall cohort and in the three different patient subgroups (patients receiving endocrine monotherapy, endocrine-based therapy, or chemotherapy) is shown. The detailed values for each subgroup, standard deviations, and significant *p*-values when comparing the results between the three different patient subgroups are shown in the corresponding **Table 4**.

The overall level of agreement for the different therapy management tools (diaries, calendars, smartphone apps, or “other”) was low among all patients surveyed in this study, and the respective mean agreement scores for each subgroup are shown in **Table 4** and **Figure 1**. Diaries were not used at all by the patients in the study.

**Table 4:** Patient characteristics and preferences regarding therapy accompaniment.

	Overall cohort (*n* = 42)	Endocrine monotherapy (*n* = 11)	Endocrine-based therapy (*n* = 17)	Chemotherapy (*n* = 14)	*p*-value comparing subgroups
Agreement with various therapy management tools					
Diary	4 (0.0)^40^	4 (0.0)^11^	4 (0.0)^15^	4 (0.0)^14^	1.0
Calendar	3.3 (1.2)^42^	4 (0.0)^11^	2.9 (1.4)^17^	3.2 (1.3)^14^	0.059
Smartphone app	3.1 (1.4)^41^	3.1 (1.3)^11^	3.1 (1.4)^16^	3.1 (1.4)^14^	0.986
Other:	3.2 (1.3)^38^	2.7 (1.5)^11^	3.4 (1.2)^15^	3.3 (1.4)^12^	0.421
Namely:		Daily notes (*n* = 2), blisters (*n* = 2), routine (*n* = 1)	Daily notes (*n* = 1), routine (*n* = 2)	Blisters (*n* = 3)	
Therapy is compatible with…					
Daily life	1.6 (0.6)^42^	1.2 (0.4)^11^	1.5 (0.5)^17^	1.9 (0.7)^14^	0.016*
Leisure plans	2.0 (1.0)^42^	1.6 (0.9)^11^	1.7 (0.8)^17^	2.6 (1.0)^14^	0.006*
Vacations	2.6 (1.1)^41^	2.1 (0.8)^11^	2.3 (1.0)^16^	3.2 (1.1)^14^	0.021*
Work	2.4 (1.2)^26^	2.5 (2.1)^2^	2.1 (1.2)^13^	2.7 (1.1)^11^	0.419
Agreement with various sources of information (for general questions)					
Outpatient oncology unit	1.8 (1.0)^41^	2.4 (1.0)^11^	1.5 (0.8)^16^	1.6 (1.1)^14^	0.035*
Internet	2.3 (1.2)^40^	2.4 (1.1)^11^	2.2 (1.2)^15^	2.3 (1.2)^14^	0.870
Patient advocacy groups	3.6 (0.9)^39^	4 (0.0)^11^	3.7 (0.6)^14^	3.2 (1.3)^14^	0.150
Group chats	3.7 (0.8)^39^	4 (0.0)^11^	3.8 (0.4)^14^	3.3 (1.3)^14^	0.155
Other patients	2.9 (1.0)^39^	2.8 (0.9)^11^	2.7 (1.0)^14^	3.1 (1.2)^14^	0.588
Emergency department	3.9 (0.5)^40^	4 (0.0)^11^	3.7 (0.8)^15^	3.9 (0.4)^14^	0.440
**Wish to have a constant contact person**	1.4 (0.5)^42^	1.6 (0.5)^11^	1.4 (0.6)^17^	1.3 (0.5)^14^	0.440
His/her qualification should Physician Nurse specialized in oncology	1.1 (0.3)^42^ 1.3 (0.7)^42^	1.3 (0.5)^11^ 1 (0.0)^11^	1.1 (0.3)^17^ 1.3 (0.8)^17^	1 (0.0)^14^ 1.4 (0.9)^14^	0.119 0.280

For each option, the mean rating on a four-point Likert scale (1 = strongly agree, 2 = partially agree, 3 = partially disagree, 4 = strongly disagree) and the standard deviation (in parentheses) are shown in the overall cohort (gray background) and in the three different patient subgroups (patients on endocrine monotherapy, endocrine-based therapy, or chemotherapy). Asterisks and bold type indicate significant p-values (from the Kruskal–Wallis test) when comparing the results between the three different patient subgroups. Superscript numbers indicate the number of patients (*n*) who answered the respective question (missing responses were common). The mean agreement scores are visualized in the corresponding **Figure 1**.

Patients on endocrine therapy were most likely to choose “other” therapy management tools (mean agreement score 2.7) to accompany their therapy, such as taking notes, counting blisters, or calling it just a “daily routine” to take their pills. Patients on endocrine-based therapy had the highest support for the use of calendars among all tools (mean agreement score 2.9), with a difference of borderline significance (*p* = 0.059) compared with the other subgroups. Patients receiving chemotherapy were unlikely to use any of the tools, with no relevant differences between the different tools (**Table 4**, **Figure 1**).

##### 3.2.1.2 Compatibility of therapy

The overall cohort reported a high level of compatibility of the therapy with daily life (mean agreement score 1.6) and with leisure plans (2.0). Compatibility with vacation (2.4) or work (2.6) was lower (**Table 4**, **Figure 1**).

Patients on endocrine therapy showed even higher compatibility of the therapy with daily life (1.2) and leisure plans (1.6) than the overall cohort and showed the lowest compatibility with work (2.5, limited data, question answered by only two patients). Patients on endocrine-based therapy reported slightly lower but still high compatibility of their therapy with daily life (1.5) and leisure plans (1.7) and sufficient compatibility with work (2.1). Patients receiving chemotherapy reported different results: they reported significantly lower compatibility with daily life (1.9, *p* = 0.016) and leisure plans (2.6, *p* = 0.006). They also reported low compatibility with vacations (3.2) or work (2.7) (**Table 4**, **Figure 1**).

##### 3.2.1.3 Sources of information

Regarding sources of information for general questions (**Table 4**, **Figure 1**), direct contact with the oncologist received the highest agreement score in the general cohort (mean agreement score 1.8), followed by searching the Internet (mean agreement score 2.3). Contacting the emergency department, patient advocacy groups, or group chats were unlikely to be used in all three subgroups (**Table 4**, **Figure 1**).

Patients on endocrine therapy reported only the outpatient oncology unit (mean agreement score 2.4), the Internet (2.4), or other patients (2.8) as sources of information for general questions—all with comparable agreement scores (**Table 4**). Patients receiving endocrine-based therapy, on the other hand, strongly preferred contacting the outpatient oncology unit (1.5, *p* = 0.035), followed by the Internet (2.2), and then other patients (2.7). Similarly, patients receiving chemotherapy primarily contacted the outpatient oncology unit (1.6) or the Internet (2.3) as a source of information (**Table 4**, **Figure 1**).

Almost all patients surveyed in this study (regardless of the treatment regimen) stated that it was important for the person responsible for their oncological therapy to remain the same (mean agreement score 1.4). The qualification of this contact person seems to be less important: both the qualification as a physician (mean agreement score 1.1) and as a specialized nurse (mean agreement score 1.3) received high scores without significant differences between the patient subgroups (**Table 4**, **Figure 1**).

#### 3.2.2 Patient preferences regarding treatment regimen

**Figure 2:**
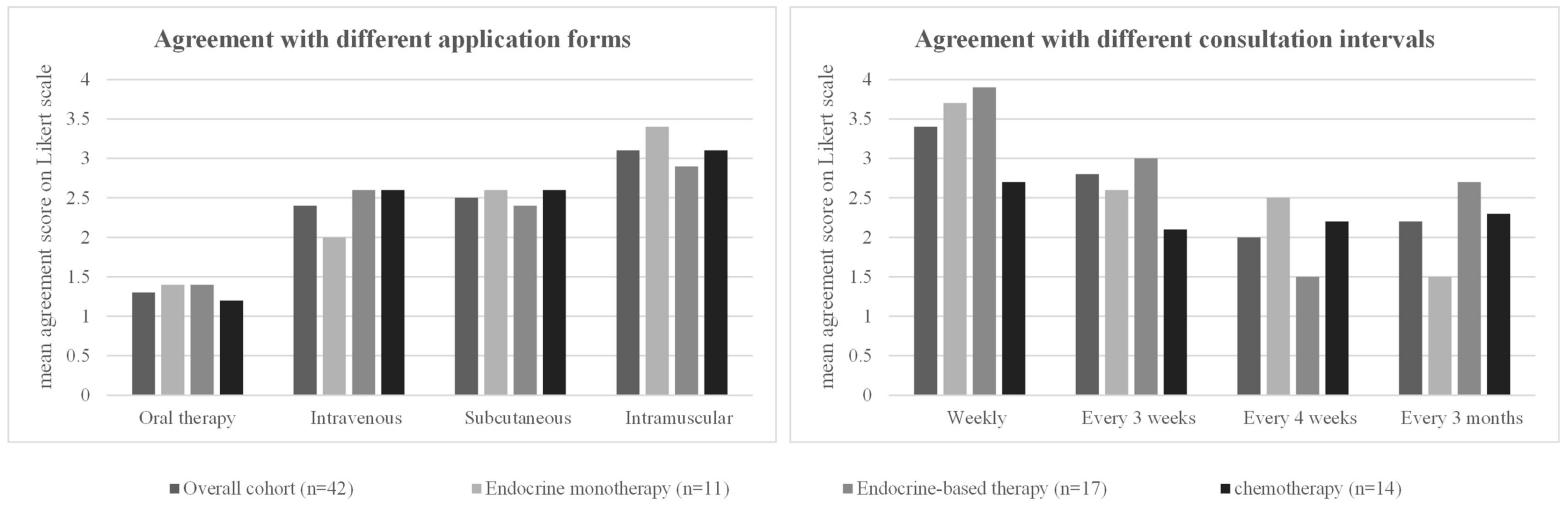
Patient preferences regarding treatment regimen. For each option, the mean rating on a four-point Likert scale (1 = strongly agree, 2 = partially agree, 3 = partially disagree, 4 = strongly disagree) in the overall cohort and in the three different patient subgroups (patients receiving endocrine monotherapy, endocrine-based therapy, or chemotherapy) is shown. The detailed values for each subgroup, standard deviations, and significant *p*-values when comparing the results between the three different patient subgroups are shown in the corresponding **Table 5**.

Regardless of the form of therapy currently being administered, all patients preferred oral tumor therapy over other forms of application [mean agreement score 1.3, *p* < 0.001 *vs*. each of the other options (intravenous, subcutaneous, and intramuscular)]. Oral therapy was followed by intravenous and subcutaneous therapy (mean agreement scores of 2.4 and 2.5, respectively). These mean agreement scores in the overall cohort were comparable to the agreement scores in each therapy subgroup (endocrine therapy *vs*. endocrine-based *vs*. chemotherapy). There were no significant differences in the preferred therapy form between the three patient subgroups (**Table 5**, **Figure 2**).

**Table 5:** Patient preferences regarding treatment regimen.

	Overall cohort (*n* = 42)	Endocrine monotherapy (*n* = 11)	Endocrine-based therapy (*n* = 17)	Chemotherapy (*n* = 14)	*p*-value comparing subgroups
Agreement with different application forms					
Oral therapy Intravenous Subcutaneous Intramuscular	1.3 (0.5)^42^ 2.4 (0.9)^40^ 2.5 (0.6)^39^ 3.1 (0.7)^39^	1.4 (0.5)^11^ 2.0 (0.9)^11^ 2.6 (0.7)^11^ 3.4 (0.7)^11^	1.4 (0.5)^17^ 2.6 (0.7)^15^ 2.4 (0.6)^14^ 2.9 (0.9)^14^	1.2 (0.4)^14^ 2.6 (1.0)^14^ 2.6 (0.6)^14^ 3.1 (0.6)^14^	0.503 0.135 0.536 0.263
Agreement with different consultation intervals					
Weekly Every 3 weeks Every 4 weeks Every 3 months	3.4 (0.9)^38^ 2.8 (0.9)^38^ 2.0 (0.9)^41^ 2.2 (1.2)^39^	3.7 (0.5)^11^ 3.6 (0.5)^11^ 2.5 (0.9)^11^ 1.5 (0.9)^11^	3.9 (0.4)^13^ 3 (0.8)^13^ 1.5 (0.8)^16^ 2.7 (1.1)^14^	2.7 (1.1)^14^ 2.1 (0.9)^14^ 2.2 (0.8)^14^ 2.3 (1.1)^14^	0.001*<0.001*0.005*0.026*

For each option, the mean rating average on a four-point Likert scale (1 = strongly agree, 2 = partially agree, 3 = partially disagree, 4 = strongly disagree) and the standard deviation (in parentheses) are shown in the overall cohort (gray background) and in the three different patient subgroups (patients receiving endocrine monotherapy, endocrine-based therapy, or chemotherapy). Asterisks and bold type indicate significant *p*-values (from the Kruskal–Wallis test) when comparing the results between the three different patient subgroups. Superscript numbers indicate the number of patients (*n*) who answered the respective question (missing responses were common). The mean agreement scores are visualized in the corresponding **Figure 2**.

A 4-week interval between therapy visits received the highest agreement score (2.0, *p* = 0.015 *vs*. 3 weeks) in the overall cohort, and weekly visit intervals were the least approved of the options given (mean agreement score 3.4, *p* < 0.001 *vs*. 3 weeks) (**Table 5**, **Figure 2**).

In the subgroup analysis, patients on endocrine monotherapy showed very high support for 3-monthly visit intervals (mean agreement score 1.5, *p* = 0.026 *vs*. the other subgroups). Weekly (3.7) or 3-weekly (3.6) consultations were least supported by the patients (**Table 5**, **Figure 2**). Patients receiving endocrine-based therapy preferred a 4-week interval between their therapy visits (mean agreement score 1.5, *p* = 0.005 compared with the other subgroups), with the least approval for weekly (3.9) consultations. Patients receiving chemotherapy had similar approval scores for 3-weekly (2.1), 4-weekly (2.2), or 3-monthly (2.3) intervals. They had the lowest agreement score for weekly consultations of all options (2.7), but this score for weekly consultations was still significantly higher than in the other two subgroups (*p* = 0.001; **Table 5**, **Figure 2**).

#### 3.2.3 Patient preferences regarding side effects

##### 3.2.3.1 Accompaniment in the event of side effects

**Figure 3:**
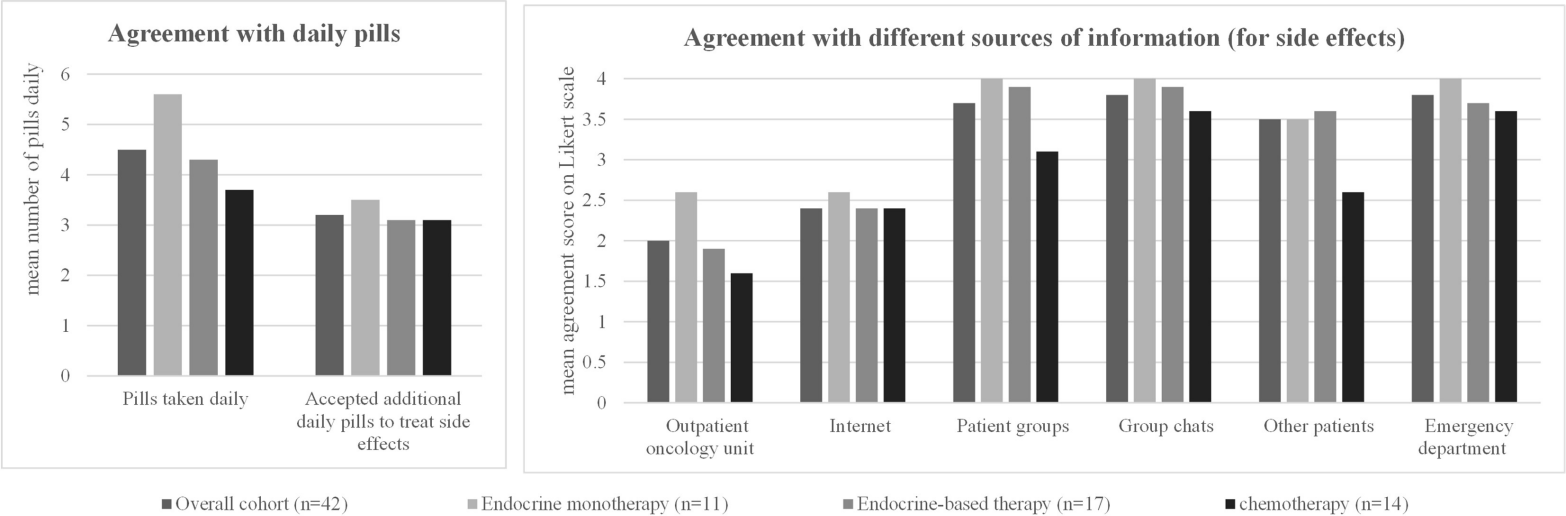
Patient characteristics and preferences regarding therapy accompaniment: for each option, the mean rating on a four-point Likert scale (1 = strongly agree, 2 = partially agree, 3 = partially disagree, 4 = strongly disagree) in the overall cohort and in the three different patient subgroups (patients receiving endocrine monotherapy, endocrine-based therapy, or chemotherapy) is shown. The detailed values for each subgroup, standard deviations, and significant *p*-values when comparing the results between the three different patient subgroups are shown in the corresponding **Table 6**.

All patients showed a high willingness to take additional medication to treat side effects even though they were already taking an average of 4.5 pills/day as co-medication (**Table 6**, **Figure 3**). Patients in the overall cohort would accept an average of 3.2 additional pills to reduce side effects. The number of additional pills accepted in the three subgroups was comparable to the overall cohort, with no significant differences between the subgroups (**Table 6**, **Figure 3**).

**Table 6:** Patient preferences regarding side effects.

	Overall cohort (*n* = 42)	Endocrine monotherapy (*n* = 11)	Endocrine-based therapy (*n* = 17)	Chemotherapy (*n* = 14)	*p*-value comparing subgroups
**A) Pills taken daily**^a^	4.5 (2.5)^42^	5.6 (1.4)^11^	4.3 (2.7)^17^	3.7 (2.7)^14^	0.071
**B) Accepted additional daily pills to treat side effects**^b^	3.2 (1.7)^42^	3.5 (1.4)^11^	3.1 (2.0)^17^	3.1 (1.8)^14^	0.659
Agreement with different sources of information (for side effects)					
Outpatient oncology unit	2.0 (1.2)^41^	2.6 (1.2)^11^	1.9 (1.1)^16^	1.6 (1.1)^14^	0.082
Internet	2.4 (1.2)^40^	2.6 (1.2)^11^	2.4 (1.2)^15^	2.4 (1.4)^14^	0.865
Patient groups	3.7 (0.8)^39^	4 (0.0)^11^	3.9 (0.0)^14^	3.1 (1.2)^14^	0.008*
Group chats	3.8 (0.5)^39^	4 (0.0)^11^	3.9 (0.5)^14^	3.6 (0.6)^14^	0.096
Other patients	3.5 (0.8)^39^	3.5 (1.0)^11^	3.6 (0.8)^14^	3.6 (0.7)^14^	0.971
Emergency department	3.8 (0.7)^40^	4 (0.0)^11^	3.7 (0.8)^15^	3.6 (0.9)^14^	0.283

A) and B): The number of pills (mean, standard deviation) is shown. C): For each option, the mean rating on a four-point Likert scale (1 = strongly agree, 2 = partially agree, 3 = partially disagree, 4 = strongly disagree) and the standard deviation (in parentheses) are shown. Results are shown for the overall cohort (grey background) and in the three different patient subgroups (patients receiving endocrine monotherapy, endocrine-based therapy, or chemotherapy). Asterisks and bold type indicate significant *p*-values (from the Kruskal–Wallis test) when comparing the results between the three different patient subgroups. Superscript numbers indicate the number of patients (*n*) who answered the respective question (missing responses were common). The mean number and mean agreement scores are visualized in the corresponding **Figure 3**.

a Patients were asked the question: “How many pills do you take in total per day?”

b Patients were asked the question: “If you could reduce the side effects of anti-tumor therapy by taking additional pills, how many would you be willing to take each day?”

In the event of side effects, most patients in the overall cohort consult the outpatient oncology clinic (mean agreement score 2.0) or the Internet (2.4). Other sources (patient groups, group chats, other patients, emergency department) were very unlikely to be contacted for side effects (**Table 6**, **Figure 3**).

In the subgroup analysis, patients on endocrine therapy were equally likely to contact the outpatient oncology clinic (2.6) and the Internet (2.6). Patients on endocrine-based therapy showed the highest agreement scores for the outpatient oncology unit (1.9) and the Internet (2.4) as a contact for side effects. Similar results were obtained for patients receiving chemotherapy, with a score of 1.6 for outpatient oncology and 2.4 for the Internet (**Table 6**, **Figure 3**).

##### 3.2.3.2 Preferred treatment regimen in case of side effects

Specifically for oral therapies, patients were asked whether they would generally prefer a continuous regimen to a “21 days on–7 days off” (21/7) regimen or vice versa. For the overall cohort, patients did not prefer one regimen over the other (47.6% *vs*. 52.4%). However, there was a significant difference when analyzing for correlations between patient subgroups and regimen choice (*p* = 0.023): patients on endocrine monotherapy preferred a continuous regimen (81.8% *vs*. 18.2%), while most patients on endocrine-based therapies preferred a 21/7 regimen (29.6% *vs*. 70.6%). Patients receiving chemotherapy did not show any clear preference for either therapy regimen (42.9% *vs*. 57.1%).

**Figure 4:**
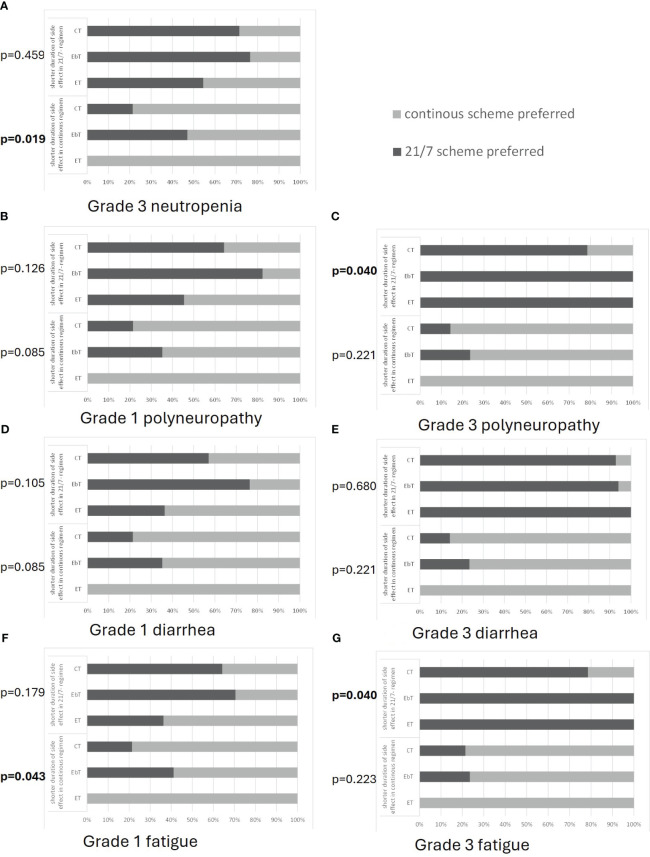
Patient preferences for either a 21-day on/7-day off regimen (21/7) or a continuous regimen with respect to the different patient groups of patients undergoing chemotherapy (CT), endocrine-based therapy (EbT), and endocrine monotherapy (ET). Each side effect [shown in **(A–G)**] could hypothetically occur for 2 days in the 21/7 regimen and for 7 days in the continuous regimen (upper part of each graph) or vice versa (lower part of each graph) Preferences for grade 3 neutropenia **(A)** (grade 1 not evaluated), in case of polyneuropathy **(B, C)**, diarrhea **(D, E)**, and fatigue **(F, G)** are shown. *p*-values indicate the result of a chi-squared test analyzing the statistical dependencies of the categorical variables.

In addition, it was examined in detail which oral therapy regimen (21/7 *vs*. continuous) is preferred by patients when the therapy regimen is associated with different side effects of varying severity (**Figure 4**). To answer this question, patients were asked to choose between two hypothetical drugs. Both were described as having the same efficacy and differed only in the therapy regimen (21/7 *vs*. continuous) and the intensity of different side effects. The side effects such as neutropenia CTCAE grade 3 with subsequent increased risk of infection (a), polyneuropathy [grade 1 (b) or grade 3 (c)], diarrhea [grade 1 (d) or grade 3 (e)], and fatigue [grade 1 (f) or grade 3 (d)] could hypothetically occur 2 or 7 days in a 28-day period and with both therapy regimens.

The results of this evaluation regarding different side effects are summarized to provide an overview of the trends in treatment decisions. The choice of a treatment regimen for each side effect for each subgroup is shown in **Figure 4**.

Patients on endocrine monotherapy generally preferred the continuous regimen, despite the presence of side effects: 100% would choose a continuous regimen if it was the regimen with a shorter duration of side effects, regardless of their intensity. Only 35%–55% would choose a 21/7 regimen if it was the regimen with a shorter duration of CTCAE grade 1 side effects (neutropenia: grade 3). The remainder would still choose a continuous regimen even at the cost of a longer duration of side effects. However, if the intensity of side effects was CTCAE grade 3 and occurred for a shorter period with the 21/7 regimen, 100% would choose the 21/7 regimen (**Figure 4**).

Patients on endocrine-based therapy, on the other hand, have a high preference for 21/7: only approximately 55%–65% would choose the continuous regimen if it was the regimen with a shorter duration of CTCAE grade 1 side effects (neutropenia: grade 3). The remainder would still choose 21/7 even at the cost of a longer duration of side effects. If side effects occurred at an intensity of CTCAE grade 3 and for a shorter period of time with the continuous regimen, approximately 75% would choose the continuous regimen. However, if 21/7 was the regimen with fewer side effects, as many as 70%–100% would choose this regimen, regardless of side effect intensity. These values were comparable across all side effects compared (**Figure 4**).

Patients undergoing chemotherapy generally chose the therapy regimen where the side effects occurred for a shorter period, but they had a tendency to favor the continuous regimen. If the continuous regimen was the regimen with a shorter period of side effects, approximately 80% would choose it, regardless of the intensity of side effects. If the 21/7 regimen was the regimen with fewer side effects, approximately 55%–65% would choose it for side effects of CTCAE grade 1 (neutropenia: grade 3) and 80–90% for side effects of CTCAE grade 3 (**Figure 4**).

### 3.3 Practitioner preferences

**Figure 5:**
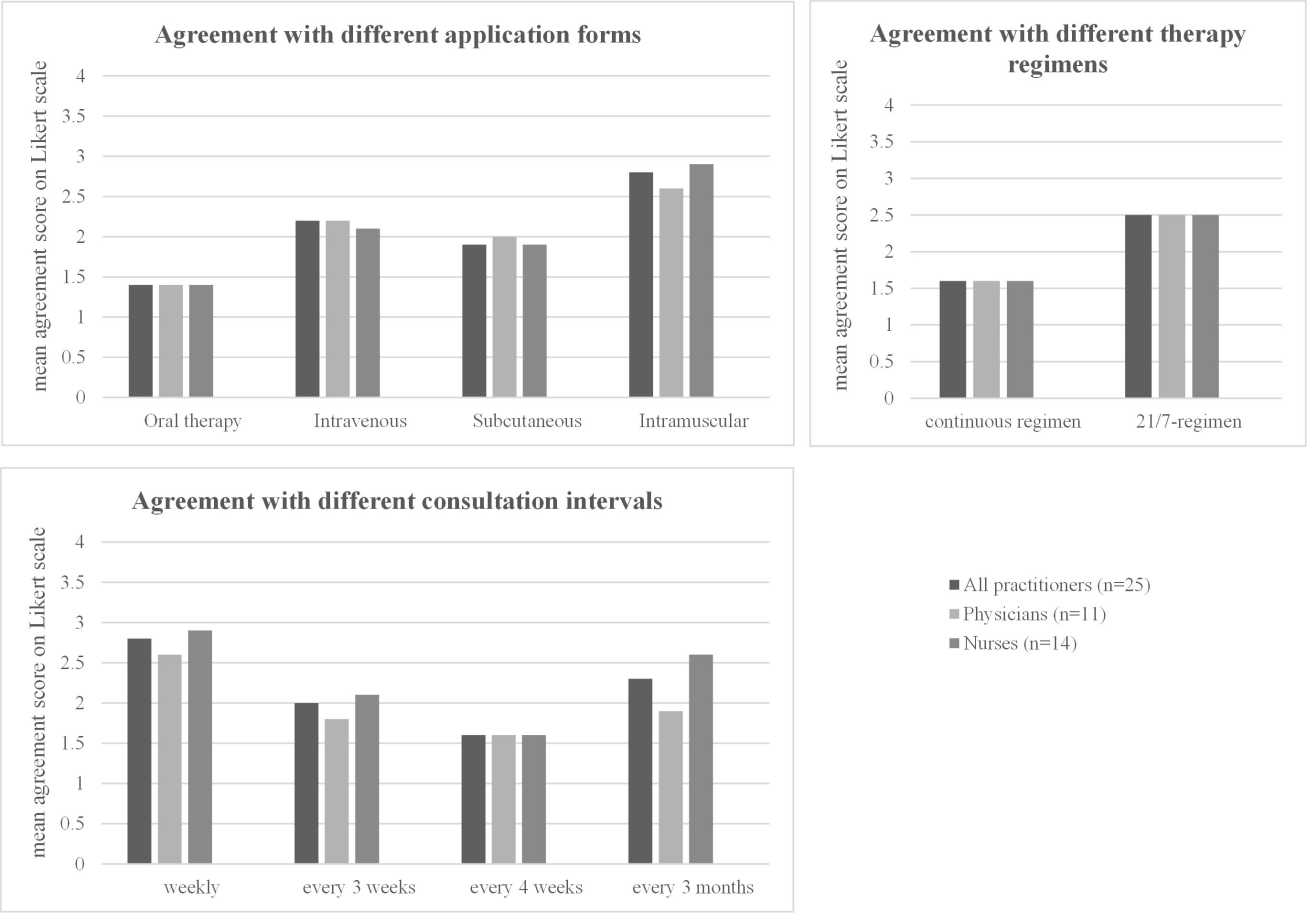
Practitioner preferences regarding the treatment regimen. For each option, the mean rating on a four-point Likert scale (1 = strongly agree, 2 = partially agree, 3 = partially disagree, 4 = strongly disagree) in the overall cohort and in the subgroups of nurses and physicians is shown. The detailed values for each subgroup, standard deviations, and *p*-values when comparing the results between the subgroups are shown in the corresponding **Table 7**.

Similar to the results obtained from patients, practitioners show a high preference for oral therapies (mean agreement score 1.4), while intramuscular injections were least preferred (2.8) (**Table 7**, **Figure 5**). Most practitioners prefer to see their patients every 3 weeks (mean agreement score 2.0) or 4 weeks (score 1.6) (**Table 7**, **Figure 5**). When asked specifically about the two different regimens for oral therapy, practitioners strongly prefer the continuous regimen (mean agreement score 1.6) over the 21/7 regimen (mean agreement score 2.5). Both physicians and nurses have similar preferences for treatment regimen and consultation intervals—there were no significant differences detectable between these two subgroups (**Table 7**, **Figure 5**).

**Table 7:** Practitioner preferences regarding treatment regimen.

	All practitioners (*n* = 25)	Physicians (*n* = 11)	Nurses (*n* = 14)	*p*-value
Agreement with different application forms				
Oral therapy Intravenous Subcutaneous Intramuscular	1.4 (0.5) 2.2 (0.8) 1.9 (0.8) 2.8 (1.0)	1.4 (0.5) 2.2 (0.9) 2.0 (0.8) 2.6 (0.9)	1.4 (0.5) 2.1 (0.8) 1.9 (0.9) 2.9 (1.0)	0.974 0.861 0.642 0.491
Agreement with different consultation intervals				
Weekly Every 3 weeks Every 4 weeks Every 3 months	2.8 (0.9) 2.0 (0.8) 1.6 (0.6) 2.3 (1.0)	2.6 (0.8) 1.8 (0.8) 1.6 (0.5) 1.9 (0.7)	2.9 (1.0) 2.1 (0.8) 1.6 (0.6) 2.6 (1.1)	0.301 0.431 0.756 0.103
Agreement with different therapy regimens				
…Continuous regimen …21/7 regimen	1.6 (0.5) 2.5 (0.7)	1.6 (0.5) 2.5 (0.7)	1.6 (0.5) 2.5 (0.8)	0.899 0.754

For each option, the mean rating on a four-point Likert scale (1 = strongly agree, 2 = partially agree, 3 = partially disagree, 4 = strongly disagree) and the standard deviation (in parentheses) are shown in the overall cohort (gray background) and in the subgroups of nurses and physicians. *p*-values (from the Kruskal–Wallis test) when comparing the results between the subgroups are shown. The mean agreement scores are visualized in the corresponding **Figure 5**.

## 4 Discussion

Oral targeted antitumor therapies with CDK4/6 inhibitors have revolutionized the treatment of HR+ HER2− metastatic breast cancer. In addition to their convincing oncological efficacy, these therapies offer numerous advantages in terms of treatment management for patients and practitioners. For example, frequent visits to the oncologist for long, time-consuming intravenous chemotherapy sessions can be avoided, and therapy can be more easily integrated into daily life. However, oral therapies require a high degree of patient responsibility, especially when it comes to managing side effects. This poses a challenge for pretherapeutic patient education. In addition, the oncology team must provide continuous oncologic care during therapy with various options for contact and information. Furthermore, interprofessional cooperation between physicians and nurses inside and outside the hospitals is necessary. As early as 1997, Liu G. et al. were able to show that patients with advanced cancer prefer oral antitumor therapy to intravenous chemotherapy if it does not compromise efficacy (23). The reasons for this choice included personal problems with intravenous lines and better compatibility with daily life by administering oral therapy in the home environment. A similar conclusion was reached by Eek D. et al. in a literature search of a total of 14 publications on patient preference for oral *vs*. intravenous administration of antitumor therapies (24). The advantages of oral therapies were seen in terms of the concrete therapeutic regimen (e.g., daily intake at home vs. weekly visits to the oncologist) and specific side effects. At the virtual San Antonio Breast Cancer Symposium in December 2020, Jaisle et al. presented the results of their online survey on expectations and preferences for oral *vs*. intravenous chemotherapy in patients with metastatic breast cancer. Assuming equal efficacy of both treatments, most respondents indicated a preference for oral (72%) over intravenous (11%) chemotherapy. The most frequently cited advantages of oral chemotherapy were ease of drug administration at home (76%), fewer appointments at the treatment center (81%), and better compatibility with work or leisure time (73%). The survey also assessed patients’ tolerance of various adverse events of varying severity caused by oral chemotherapy. Respondents were least willing to tolerate adverse events such as hand-foot syndrome, diarrhea, neuropathy, and nausea with a severity of CTCAE grade III/IV (25).

Similar results regarding treatment regimen preferences were obtained in the real-world survey conducted as part of this work. All patients, whether they had previous experience with oral therapies (patients currently receiving endocrine therapy or endocrine-based therapy) or not (patients currently receiving chemotherapy), strongly preferred oral therapy when asked for their approval of different therapy regimens. Patients currently receiving endocrine-based therapy reported that therapy was more compatible with daily life, leisure time, and vacations than patients receiving chemotherapy. This is encouraging, because in a study of patients with metastatic breast cancer that did not focus specifically on treatment regimens, more than half reported that their disease had “very much” affected their family’s well-being, and one-fifth reported that it had strongly affected their responsibilities and social life (26). In our survey, endocrine-based therapy was also reported to be highly compatible with work. This is important because women with metastatic breast cancer who could continue to work had a better quality of life than those who could not (26). This is probably one of the greatest benefits of oral antitumor therapy that we were able to could confirm in our study: the drugs are taken at home, independent of a clinical setting, and can be flexibly integrated into a person’s daily routine.

The different oral CDK4/6 inhibitors differ in terms of side effects and regimen between continuous intake and 21-day intake followed by a 7-day break. Our study does not provide a clear indication as to whether patients prefer a particular dosing regimen for oral antitumor therapy: 47.6% of the respondents chose the 21/7 regimen and 52.4% the continuous regimen. Looking at different patient groups, it becomes clear that patients are most likely to vote for the regimen they are already familiar with: patients on endocrine monotherapy voted predominantly for the continuous regimen. Patients on endocrine-based therapy (with a high proportion of palbo-/ribociclib-experienced patients) voted for the 21/7 regimen and patients on chemotherapy—unfamiliar with either regimen—voted approximately 50/50%. Practitioners, on the other hand, strongly favored the continuous regimen, probably because it appears to be easier to manage. When the therapy regimens were associated with specific side effects of varying severity, most of the respondents chose the regimen in which the adverse event occurred for a shorter period of time, e.g., only 2 days per month instead of 7 days per month. However, patients on endocrine monotherapy still tended to stick with the continuous regimen and patients on endocrine-based therapy tended to stick with the 21/7 regimen—even if this was the regimen in which the side effect lasted longer. This was true for mild CTCAE grade 1 side effects (diarrhea, fatigue, polyneuropathy) and also for CTCAE grade 3 neutropenia. This result again reflects the tendency of patients to prefer a treatment regimen with which they are already familiar. However, when the side effect was severe (CTCAE grade 3), the influence of “habit” became less important: patients generally chose the treatment regimen in which the side effect occurred for a shorter period of time, regardless of whether it was a continuous or a 21/7 regimen.

In general, the establishment of a continuous and intimate relationship between practitioners and patients, as well as regular appointments with the possibility of needs-based therapy support, can contribute significantly to increase adherence to oral antitumor therapy. Regarding therapy accompaniment, we might assume certain wishes of our patients, but we should analyze scientifically what is really important to our patients, to establish tools, personal contact, and visits preferred and to abandon those not much used.

In our study, most patients preferred to see their oncologist every 4 weeks. In general, patients receiving endocrine monotherapy and endocrine-based therapy were more comfortable with longer intervals between visits than those currently receiving chemotherapy. The general trend of highest support for a visit frequency of approximately once a month (every 3 or 4 weeks) is also reflected in the practitioners’ survey. They also seem to have the best experience with these consultation intervals and do not want to see their patients less often, even though this would further reduce their workload—presumably, regular check-ups are still necessary for them to monitor the therapy. There were no differences between physicians and nurses. It also seems to be important for all patients, regardless of their treatment regimen, to have a constant contact person in the outpatient oncology setting, who may be either a physician or a specialized nurse. Specialized nurses are an adaptation that most oncology facilities have already implemented to improve therapy accompaniment. All breast centers in Germany already work with breast care nurses, and some also employ advanced practice nurses (APNs). APNs are academically qualified nurses, who offer specialized, nurse-led consultations, with extensive pretreatment discussions and close monitoring during therapy (27). However, in a recent UK study, only 56% of patients with metastatic breast cancer had access to a specialized nurse (26). A further development to provide patients with the best possible support throughout their treatment is the patient/onco-coach treatment model. Onco-coaches are the central link between the physician and the patient. They provide support and education to patients throughout the entire treatment process and act as a personal advisor. In addition, they collect feedback from patients and pass it on in a targeted manner (28). Onco-coaches are not yet common in Germany.

In-depth patient education is one of the central aspects of oral antitumor therapy. Regular personal training sessions on the individual drugs, interactions, side effects, prophylaxis, and behavior in everyday life can increase patients’ knowledge, especially about oral tumor therapies, and help to promote motivation and self-management. A recent survey of metastatic breast cancer patients reported that information is very important to the patients: 71% of all patients in this study reported that they wished they had known more about metastatic breast cancer before their diagnosis and 47% reported that they still do not fully understand their disease (26). In addition to treatment management, breast cancer patients also have some very specific information needs, such as information about sexual health (29). Even 15 months after diagnosis, breast cancer patients report unmet needs (30).

It is likely that patient education should be more in-depth for patients undergoing endocrine-based therapies compared with traditional intravenous chemotherapy, where patients receive their medications and co-medications in a controlled medical setting. In our survey, outpatient oncology consultations received the highest agreement scores as sources of information, regardless of treatment regimen and for both general questions and side effects. However, the Internet is also used as a source of information (also reflected in high agreement scores). A recent study of newly diagnosed breast cancer patients found similar results: in this study, physicians and nurses were the most important sources of information, closely followed by the Internet which was used by 81% of all patients in this study (31). Numerous other studies also report the use of the Internet as an important source of information (26, 32). These findings highlight the importance of developing evidence-based websites and online information tools for patients to obtain reliable, peer-reviewed information and to share these web-based information resources with patients during face-to-face consultations. This will contribute significantly to patient education and will prevent patients from receiving unfiltered and even false information from various not well-controlled websites. Various tools and websites already exist, e.g., from patient support groups or oncology societies. In Germany, new legislation allows for the prescription of validated eHealth tools: the digital coach “PINK!” contributes to patient education through coaching modules. Patients receive pseudo-individualized information, practical tips, instructions, and tutorials in the areas of exercise, nutrition, and mental health (33).

Additional tools can be helpful not only for patient education but also for treatment management (34). Traditional tools include patient diaries or calendars that record, for example, pill intake, side effects and complaints, and upcoming appointments. However, all these tools received rather low approval rates in our study. For some time now, eHealth-based therapy support has been available, such as the CANKADO app with artificial intelligence-based individualized support to reduce severe side effects and increase adherence. CANKADO reliably reminds patients of upcoming pills or other medication-specific tasks, such as blood glucose measurements, and offers the possibility to document daily symptoms. The recorded health status can serve as a basis for the next discussion with the attending physician. The goal is to conserve resources on the part of care providers (staff, time) and patients (independence, time) (22). It has been shown that the use of CANKADO in metastatic breast cancer patients can prolong the time to quality of life deterioration (35). The use of the app received the highest agreement scores of all therapy management tools in our survey but with a still rather low mean agreement score of 3.1 in the overall cohort. This score was also not significantly higher in patients on endocrine-based therapy, for whom it could be a helpful tool for self-management of their therapy. Regular support and assistance from a specially trained caregiver like an APN or an onco-coach may increase the number of users and thus improve adherence in patients with oral antitumor therapy.

Our study represents an important real-world experience of patients with breast cancer, but several limitations should still be considered. The total number of patients—with 42 patients divided into three subgroups—is quite small. However, our study still provides a representative sample of breast cancer patients treated in oncology centers today. Comparable studies, analyzing, e.g., the information-seeking behavior of breast cancer patients, achieve comparable patient numbers and come to comparable results (31). Therefore, we were able to present qualitative data on the important wishes and needs of these patients and can also provide important subgroup analyses.

As the questionnaires were designed specifically for this study, we cannot provide a validated survey. However, it is difficult to obtain such individual and subjective information in a validated and rigid survey. Often, such valuable information is provided in patient workshops, so that qualitative surveys are already an improvement over pure workshops (36). Another limitation may be that the different CDK4/6 inhibitors, which were grouped under the term “endocrine-based therapy” in our study, have different treatment regimens and side effects, which may also influence the needs reported by patients. However, the limited number of patients in this subgroup (*n* = 17) would have resulted in very small patient numbers if each CDK4/6 inhibitor were considered in isolation. This analysis addresses the overall difference between CDK4/6 inhibitors and chemotherapy or endocrine monotherapy. Even if the CDK4/6 inhibitors have different treatment regimens, the differences with completely different forms of therapy, such as intravenous chemotherapy, are likely to be much greater. The aim of this study was to identify the general differences in treatment preferences among patients undergoing endocrine-based therapy. Furthermore, our study is monocentric and center-specific effects cannot be excluded. In addition, the patient population may be somewhat biased due to a selection for more complex cases at a university hospital. Patients in smaller outpatient oncology centers with, e.g., fewer additional diagnoses, might have slightly different wishes.

In conclusion, our study showed a high desire for oral antitumor therapies among both patients and practitioners without a clear preference for a specific therapy regimen. Since oral therapies are taken by patients on their own, thorough patient education—about the individual therapy regimen, relevant side effects, and their management and potential interactions—is necessary to ensure therapy safety. Patients need to be provided with written booklets with detailed information about their therapy, which they can use to look up information at home, but which they can also take with them in case of an emergency to inform the medical team in the emergency department. Of course, they need to know all emergency contacts and phone numbers. For outpatient oncology care, patients should be seen regularly every 4 weeks, either in person or by phone or video call. Specialized nurses or additional tools such as eHealth modules can help to improve treatment management.

## Data availability statement

The raw data supporting the conclusions of this article will be made available by the authors, without undue reservation.

## Ethics statement

The studies involving humans were approved by LMU Munich ethics comittee, LMU Munich, Munich, Germany. The studies were conducted in accordance with the local legislation and institutional requirements. Written informed consent for participation was not required from the participants or the participants’ legal guardians/next of kin because the only way of gathering information on patients was by asking for them in questionnaires. Patients were informed about the survey when they received the questionnaires. When patients returned the questionnaires, this was regarded as consent to participate and therefore no separate informed consent was required according to the ethical approval.

## Author contributions

AH: Formal analysis, Validation, Visualization, Writing – original draft. FH: Data curation, Formal analysis, Investigation, Project administration, Visualization, Writing – review & editing. AD: Writing – review & editing. CS: Writing – review & editing. AK: Writing – review & editing. NH: Conceptualization, Funding acquisition, Methodology, Resources, Supervision, Writing – review & editing. RW: Conceptualization, Funding acquisition, Resources, Supervision, Validation, Writing – review & editing.

## Supplementary material

Supplementary File 1 Questionnaire for patients (in German).

Supplementary File 2 Questionnaire for practitioners (in German).

Supplementary File 3 Questionnaire for patients (translated to English for submission).

Supplementary File 4 Questionnaire for practitioners (translated to English for submission, shortened to the questions relevant for this publication).
