# Supplementary material for: What are the needs in oral antitumor therapy? An analysis of patients’ and practitioners’ preferences
Source: Front Oncol. 2024 Jun 27;14:1388087. doi: 10.3389/fonc.2024.1388087 (PMC11236681; doi:10.3389/fonc.2024.1388087)
Supplement: Supplementary File 1 — Questionnaire for patients (in German). [file DataSheet_1.docx]

Sehr geehrte Patientin, sehr geehrter Patient,

Sie erhalten derzeit zur Behandlung Ihres fortgeschrittenen Mammakarzinoms eine Medikamenten-therapie in unserer Onkologischen Tagesklinik. Wir möchten Ihre medizinische Betreuung und den Zeitaufwand Ihrer Besuche während dieser Therapie optimieren. Dazu bitten wir Sie um Ihre Mithilfe, indem Sie diesen Fragebogen ausfüllen. Darin stellen wir Ihnen demografische Fragen zu Ihrer Person, zur Ihrer Erkrankung und zu Ihrer aktuellen Therapie. Anschließend möchten wir von Ihnen wissen, wie sich diese Therapie in Ihren Alltag integrieren lässt und welche Therapien sie präferieren würden.

Die Fragebögen sind unterschiedlich farbig markiert, um Ihre Antworten Ihrer aktuellen Therapie (z.B. Chemotherapie, Antihormontherapie oder Kombinationstherapie) zuzuordnen und dementsprechend auswerten zu können. Da nur eine kleine Anzahl von Patienten(innen) befragt wird (insgesamt ca. 60), ist das Risiko einer Re-Identifikation erhöht. Ihre übermittelten Antworten werden selbstverständlich vertraulich behandelt und ohne Ihre persönlichen Daten verarbeitet. Bitte geben Sie deshalb diesen Fragebogen ausgefüllt in die dafür vorgesehenen Kästen im Stationsstützpunkt der Onkologischen Tagesklinik.

Die Pharmafirma Lilly Deutschland GmbH unterstützt dieses Projekt finanziell, um entstehende Mehrkosten abdecken zu können. Für Sie als Teilnehmer ist keine finanzielle Entschädigung mit der Beantwortung der Fragebögen vorgesehen. Nach der Auswertung werden ausschließlich komplette Datensätze, ohne personen-identifizierende Merkmale an den Sponsor übermittelt.

Mit der Abgabe des Fragebogens geben Sie Ihre Einwilligung zu unserem Projekt. Ein Widerruf und die Löschung der erhobenen Daten ist danach nicht mehr möglich.

Für Rückfragen, Anregungen und Wünsche steht PD Dr. med. Rachel Würstlein und ihr Team gerne zu Ihrer Verfügung.

Vielen Dank für Ihre Unterstützung

Prof. Dr. med. Nadia Harbeck, PD Dr. med. Rachel Würstlein und Team

Bitte beantworten Sie jede der Fragen, indem Sie die Antwortmöglichkeit auswählen, die am ehesten auf Sie zutrifft bzw. die entsprechende Antwort eintragen.

| 1. **Angaben zur Person** | | | | | | | | | | | |
| --- | --- | --- | --- | --- | --- | --- | --- | --- | --- | --- | --- |
| 1. **Geschlecht:** männlich weiblich | | | | | | 1. **Geburtsjahr:** ____________ | | | | | |
| 1. **Was ist ihr höchster Bildungsabschluss?** | | | | | | | | | | | |
| Hauptschulabschluss/Volksschule | | | | Realschulabschluss/Mittlere Reife | | | | | Abitur | | |
| Bachelor | Master/Diplom | | | | | | Promotion | | | | Habilitation |
| 1. **In welchem Umfang sind Sie beschäftigt?** | | | | | | | | | | | |
| Vollzeit | | | Teilzeit | | | | | Student/Auszubildende | | | |
| Rentner | | | ehrenamtliche Tätigkeit | | | | | unbeschäftigt | | | |
| 1. **Wie ist Ihre familiäre Situation?** | | | | | | | | | | | |
| ledig | | | geschieden | | | | | Lebenspartnerschaft aufgehoben | | | |
| verheiratet | | | verwitwet | | | | | Lebenspartner/-in verstorben | | | |
| getrennt lebend | | | Lebenspartnerschaft | | | | |  | | | |
| 1. **Haben Sie Kinder?** | | | | | | | | | | | |
| Ja Nein | | | Falls ja: Wie viele? _______________ | | | | | | | | |
| 1. **Angaben zu Ihrer Erkrankung** | | | | | | | | | | | |
| 1. **Wann wurde Ihre Erkrankung das erste Mal diagnostiziert (Jahr)?** _________________ | | | | | | | | | | | |
| 1. **Seit wann ist bekannt, dass Ihr Tumor gestreut hat (Jahr)?** _________________ | | | | | | | | | | | |
| 1. **Welche Organe sind von den Metastasen betroffen?** | | | | | | | | | | | |
| Knochen Lunge/Pleura Leber Haut Gehirn Andere: __________________ | | | | | | | | | | | |
| 1. **Angaben zu Ihrer Therapie** | | | | | | | | | | | |
| 1. **Erhalten Sie eine Therapie zur Erhaltung der Knochenstabilität (z.B. Bondronat, X-GEVA)?** | | | | | | | | | | | |
| Ja Nein | | | Falls ja: welche? _______________________ | | | | | | | | |
| 1. **Welche Tumortherapie erhalten Sie gerade?** | | | | | | | | | | | |
| eine Chemotherapie (z.B. Paclitaxel, Avastin)  eine antihormonelle Therapie (z.B. Tamoxifen®, Letrozol®, Faslodex®) | | | | | | | | | | | |
| eine Kombinationstherapie (z.B. Ibrance® oder Verzenios® mit Letrozol® oder Faslodex®) | | | | | | | | | | | |
| 1. **Erhalten Sie eine Therapie zur Unterdrückung der Eierstockfunktion (z.B. mit Zoladex®)?** | | | | | | | | | | | |
| Ja Nein | | | Falls ja: welche? _______________________ | | | | | | | | |
|  | | |  | | | | | | | | |
|  | | |  | | | | | | | | |
| 1. **Wünsche zu Ihrer Therapie** | | | | | | | | | | | |
| 1. **Wenn Sie die Wahl hätten, welche Form der Darreichung von Medikamenten würden Sie bevorzugen?** | | | | | | | | | | | |
|  | | Trifft nicht zu | | | Trifft eher nicht zu | | | Trifft eher zu | | Trifft vollkommen zu | |
| Tablette | |  | | |  | | |  | |  | |
| Spritze ins Fettgewebe | |  | | |  | | |  | |  | |
| Spritze in den Muskel | |  | | |  | | |  | |  | |
| Infusion (intravenös) | |  | | |  | | |  | |  | |
| 1. **Wie viele Tabletten nehmen Sie insgesamt an einem Tag ein?** | | | | | | | | | | | |
| 1 Tablette 2 Tabletten 3 Tabletten 4 Tabletten 5 Tabletten | | | | | | | | | | | |
| 6 Tabletten 7 Tabletten 8 Tabletten 9 Tabletten 10 Tabletten | | | | | | | | | | | |
| mehr als 10 Tabletten | | | | | | | | | | | |
| 1. **Wenn Sie Nebenwirkungen der Tumortherapie mit der Einnahme weiterer Tabletten reduzieren könnten, wie viele Tabletten würden Sie zusätzlich einnehmen?** | | | | | | | | | | | |
| 1 Tablette 2 Tabletten 3 Tabletten 4 Tabletten 5 Tabletten | | | | | | | | | | | |
| mehr als 5 Tabletten | | | | | | | | | | | |
| 1. **Benutzen Sie zur Erinnerung Ihrer Tabletteneinnahme folgende Hilfsmittel?** | | | | | | | | | | | |
|  | | Trifft nicht zu | | | Trifft eher nicht zu | | | Trifft eher zu | | Trifft vollkommen zu | |
| Tagebuch | |  | | |  | | |  | |  | |
| Kalender | |  | | |  | | |  | |  | |
| App auf dem Handy | |  | | |  | | |  | |  | |
| Andere: ____________________ | |  | | |  | | |  | |  | |
| 1. **Wenn Sie die Besuche in der Onkologischen Tagesklinik selbst bestimmen dürften, in welchen Abständen würden Sie bevorzugt kommen?** | | | | | | | | | | | |
|  | | Trifft nicht zu | | | Trifft eher nicht zu | | | Trifft eher zu | | Trifft vollkommen zu | |
| wöchentlich | |  | | |  | | |  | |  | |
| 3-wöchentlich | |  | | |  | | |  | |  | |
| monatlich | |  | | |  | | |  | |  | |
| alle 3 Monate | |  | | |  | | |  | |  | |
| 1. **Können Sie Ihre aktuelle Therapie in folgenden Lebenssituationen gut integrieren?** | | | | | | | | | | | |
|  | | Trifft nicht zu | | | Trifft eher nicht zu | | | Trifft eher zu | | Trifft vollkommen zu | |
| im Alltag | |  | | |  | | |  | |  | |
| im Berufsleben | |  | | |  | | |  | |  | |
| in der Freizeit | |  | | |  | | |  | |  | |
| bei Urlaubsreisen | |  | | |  | | |  | |  | |
| 1. **Ich wünsche mir einen kontinuierlichen Ansprechpartner für die Therapiezeit in unserer Onkologischen Tagesklinik.** | | | | | | | | | | | |
|  | | Trifft nicht zu | | | Trifft eher nicht zu | | | Trifft eher zu | | Trifft vollkommen zu | |
|  | |  | | |  | | |  | |  | |

| 1. **Dieser Ansprechpartner dürfte folgender Berufsgruppe angehören:** | | | | |
| --- | --- | --- | --- | --- |
|  | Trifft nicht zu | Trifft eher nicht zu | Trifft eher zu | Trifft vollkommen zu |
| Arzt/Ärztin |  |  |  |  |
| speziell geschultes Pflegepersonal |  |  |  |  |
| 1. **Haben Sie sich während der aktuellen Therapie schon einmal aufgrund allgemeiner Fragen an folgende Instanzen gewendet bzw. nachgelesen?** | | | | |
|  | Trifft nicht zu | Trifft eher nicht zu | Trifft eher zu | Trifft vollkommen zu |
| Internet |  |  |  |  |
| Selbsthilfegruppe |  |  |  |  |
| Chat-Gruppen |  |  |  |  |
| andere Patienten |  |  |  |  |
| Notaufnahme |  |  |  |  |
| Tagesklinik |  |  |  |  |
| 1. **Haben Sie sich während der aktuellen Therapie schon einmal aufgrund von Nebenwirkungen an folgende Instanzen gewendet bzw. nachgelesen?** | | | | |
|  | Trifft nicht zu | Trifft eher nicht zu | Trifft eher zu | Trifft vollkommen zu |
| Internet |  |  |  |  |
| Selbsthilfegruppe |  |  |  |  |
| Chat-Gruppen |  |  |  |  |
| andere Patienten |  |  |  |  |
| Notaufnahme |  |  |  |  |
| Tagesklinik |  |  |  |  |

Liebe Patientin, lieber Patient,

auf den folgenden Seiten werden mehrmals jeweils 2 Medikamente zur Tumortherapie in Tablettenform zur Behandlung von metastasiertem Brustkrebs vorgestellt. Es handelt sich dabei nicht um tatsächliche Medikamente, sondern um fiktive Medikamente, die zu Forschungszwecken erfunden wurden. Wir möchten Sie bitten, die Medikamente zu vergleichen und anzugeben, welches Medikament sie jeweils bevorzugen, wenn sie sich **ausschließlich** in den angegebenen Merkmalen unterscheiden. Sie sollen davon ausgehen, dass **beide Medikamente die gleiche Wirksamkeit** besitzen und sich ansonsten auch in allen anderen Merkmalen, außer den angegebenen, gleichen. Jede Ihrer Einschätzungen ist wichtig für uns. Bitte lassen Sie keine aus, auch wenn ein Vergleich schwierig sein mag.

Welches der beiden Medikamente würden Sie jeweils bevorzugen?

**Medikament A** nehmen Sie 1x täglich zur selben Uhrzeit ein. Die Einnahme erfolgt an 21 Tagen gefolgt von 7 Tagen Pause (21/7 Schema) und so weiter.

**Medikament B** nehmen Sie 2x täglich, also morgens und abends zur selben Uhrzeit ein. Die Einnahme erfolgt durchgehend ohne Pause.

1. Allgemeine Präferenz

|  | **Medikament A** |  | **Medikament B** |
| --- | --- | --- | --- |
| Darreichung | Tablette |  | Tablette |
| Häufigkeit der Einnahme | 1x täglich (21/7 Schema) | **ODER** | 2x täglich (ohne Pause) |
|  |  |  |  |
| Welches Medikament würden Sie wählen? (Kreuzen sie eines an) | Medikament A |  | Medikament B |

1. Nebenwirkung: Neutropenie (niedrige weiße Blutkörperchen)

|  | **Medikament A** |  | **Medikament B** |
| --- | --- | --- | --- |
| Darreichung | Tablette |  | Tablette |
| Häufigkeit der Einnahme | 1x täglich (21/7 Schema) | **ODER** | 2x täglich (ohne Pause) |
| **Häufigkeit niedriger weißer Blutkörperchen und erhöhtes Infektionsrisiko** | **7 Tage im Monat** |  | **2 Tage im Monat** |
|  |  |  |  |
| Welches Medikament würden Sie wählen? (Kreuzen sie eines an) | Medikament A |  | Medikament B |

|  | **Medikament A** |  | **Medikament B** |
| --- | --- | --- | --- |
| Darreichung | Tablette |  | Tablette |
| Häufigkeit der Einnahme | 1x täglich (21/7 Schema) | **ODER** | 2x täglich (ohne Pause) |
| **Häufigkeit niedriger weißer Blutkörperchen und erhöhtes Infektionsrisiko** | **2 Tage im Monat** |  | **7 Tage im Monat** |
|  |  |  |  |
| Welches Medikament würden Sie wählen? (Kreuzen sie eines an) | Medikament A |  | Medikament B |

1. Nebenwirkung: Missempfindungen in Händen und/oder Füßen

|  | **Medikament A** |  | **Medikament B** |
| --- | --- | --- | --- |
| Darreichung | Tablette |  | Tablette |
| Häufigkeit der Einnahme | 1x täglich (21/7 Schema) | **ODER** | 2x täglich (ohne Pause) |
| **Häufigkeit von leichten Missempfindungen in Fingerspitzen und Zehen** | **7 Tage im Monat** |  | **2 Tage im Monat** |
|  |  |  |  |
| Welches Medikament würden Sie wählen? (Kreuzen sie eines an) | Medikament A |  | Medikament B |

|  | **Medikament A** |  | **Medikament B** |
| --- | --- | --- | --- |
| Darreichung | Tablette |  | Tablette |
| Häufigkeit der Einnahme | 1x täglich (21/7 Schema) | **ODER** | 2x täglich (ohne Pause) |
| **Häufigkeit von leichten Missempfindungen in Fingerspitzen und Zehen** | **2 Tage im Monat** |  | **7 Tage im Monat** |
|  |  |  |  |
| Welches Medikament würden Sie wählen? (Kreuzen sie eines an) | Medikament A |  | Medikament B |

|  | **Medikament A** |  | **Medikament B** |
| --- | --- | --- | --- |
| Darreichung | Tablette |  | Tablette |
| Häufigkeit der Einnahme | 1x täglich (21/7 Schema) | **ODER** | 2x täglich (ohne Pause) |
| **Häufigkeit von Gefühlsstörungen, Gefühlsverlust (eingeschränkte Funktion im Alltag)** | **7 Tage im Monat** |  | **2 Tage im Monat** |
|  |  |  |  |
| Welches Medikament würden Sie wählen? (Kreuzen sie eines an) | Medikament A |  | Medikament B |

|  | **Medikament A** |  | **Medikament B** |
| --- | --- | --- | --- |
| Darreichung | Tablette |  | Tablette |
| Häufigkeit der Einnahme | 1x täglich (21/7 Schema) | **ODER** | 2x täglich (ohne Pause) |
| **Häufigkeit von Gefühlsstörungen, Gefühlsverlust (eingeschränkte Funktion im Alltag)** | **2 Tage im Monat** |  | **7 Tage im Monat** |
|  |  |  |  |
| Welches Medikament würden Sie wählen? (Kreuzen sie eines an) | Medikament A |  | Medikament B |

|  | **Medikament A** |  | **Medikament B** |
| --- | --- | --- | --- |
| Darreichung | Tablette |  | Tablette |
| Häufigkeit der Einnahme | 1x täglich (21/7 Schema) | **ODER** | 2x täglich (ohne Pause) |
| **Häufigkeit starker Missempfindungen**  **→ Einschränkung der Selbstversorgung** | **7 Tage im Monat** |  | **2 Tage im Monat** |
|  |  |  |  |
| Welches Medikament würden Sie wählen? (Kreuzen sie eines an) | Medikament A |  | Medikament B |

|  | **Medikament A** |  | **Medikament B** |
| --- | --- | --- | --- |
| Darreichung | Tablette |  | Tablette |
| Häufigkeit der Einnahme | 1x täglich (21/7 Schema) | **ODER** | 2x täglich (ohne Pause) |
| **Häufigkeit starker Missempfindungen**  **→ Einschränkung der Selbstversorgung** | **2 Tage im Monat** |  | **7 Tage im Monat** |
|  |  |  |  |
| Welches Medikament würden Sie wählen? (Kreuzen sie eines an) | Medikament A |  | Medikament B |

1. Nebenwirkung: Durchfall

|  | **Medikament A** |  | **Medikament B** |
| --- | --- | --- | --- |
| Darreichung | Tablette |  | Tablette |
| Häufigkeit der Einnahme | 1x täglich (21/7Schema) | **ODER** | 2x täglich (ohne Pause) |
| **Häufigkeit vermehrter Stuhlgänge (bis zu 1x täglich)** | **7 Tage im Monat** |  | **2 Tage im Monat** |
|  |  |  |  |
| Welches Medikament würden Sie wählen? (Kreuzen sie eines an) | Medikament A |  | Medikament B |

|  | **Medikament A** |  | **Medikament B** |
| --- | --- | --- | --- |
| Darreichung | Tablette |  | Tablette |
| Häufigkeit der Einnahme | 1x täglich (21/7Schema) | **ODER** | 2x täglich (ohne Pause) |
| **Häufigkeit vermehrter Stuhlgänge (bis zu 1x täglich)** | **2 Tage im Monat** |  | **7 Tage im Monat** |
|  |  |  |  |
| Welches Medikament würden Sie wählen? (Kreuzen sie eines an) | Medikament A |  | Medikament B |

|  | **Medikament A** |  | **Medikament B** |
| --- | --- | --- | --- |
| Darreichung | Tablette |  | Tablette |
| Häufigkeit der Einnahme | 1x täglich (21/7Schema) | **ODER** | 2x täglich (ohne Pause) |
| **Häufigkeit vermehrter Stuhlgänge (2-5x täglich)** | **7 Tage im Monat** |  | **2 Tage im Monat** |
|  |  |  |  |
| Welches Medikament würden Sie wählen? (Kreuzen sie eines an) | Medikament A |  | Medikament B |

|  | **Medikament A** |  | **Medikament B** |
| --- | --- | --- | --- |
| Darreichung | Tablette |  | Tablette |
| Häufigkeit der Einnahme | 1x täglich (21/7Schema) | **ODER** | 2x täglich (ohne Pause) |
| **Häufigkeit vermehrter Stuhlgänge (2-5x täglich)** | **2 Tage im Monat** |  | **7 Tage im Monat** |
|  |  |  |  |
| Welches Medikament würden Sie wählen? (Kreuzen sie eines an) | Medikament A |  | Medikament B |

|  | **Medikament A** |  | **Medikament B** |
| --- | --- | --- | --- |
| Darreichung | Tablette |  | Tablette |
| Häufigkeit der Einnahme | 1x täglich (21/7Schema) | **ODER** | 2x täglich (ohne Pause) |
| **Häufigkeit vermehrter Stuhlgänge (mehr als** **6 x täglich)** | **7 Tage im Monat** |  | **2 Tage im Monat** |
|  |  |  |  |
| Welches Medikament würden Sie wählen? (Kreuzen sie eines an) | Medikament A |  | Medikament B |

|  | **Medikament A** |  | **Medikament B** |
| --- | --- | --- | --- |
| Darreichung | Tablette |  | Tablette |
| Häufigkeit der Einnahme | 1x täglich (21/7Schema) | **ODER** | 2x täglich (ohne Pause) |
| **Häufigkeit vermehrter Stuhlgänge (mehr als** **6 x täglich)** | **2 Tage im Monat** |  | **7 Tage im Monat** |
|  |  |  |  |
| Welches Medikament würden Sie wählen? (Kreuzen sie eines an) | Medikament A |  | Medikament B |

1. Nebenwirkung: Abgeschlagenheit/Müdigkeit

|  | **Medikament A** |  | **Medikament B** |
| --- | --- | --- | --- |
| Darreichung | Tablette |  | Tablette |
| Häufigkeit der Einnahme | 1x täglich (21/7 Schema) | **ODER** | 2x täglich (ohne Pause) |
| **Häufigkeit leichter Müdigkeit**  **→ Besserung durch kurze Ruhepausen** | **7 Tage im Monat** |  | **2 Tage im Monat** |
|  |  |  |  |
| Welches Medikament würden Sie wählen? (Kreuzen sie eines an) | Medikament A |  | Medikament B |

|  | **Medikament A** |  | **Medikament B** |
| --- | --- | --- | --- |
| Darreichung | Tablette |  | Tablette |
| Häufigkeit der Einnahme | 1x täglich (21/7 Schema) | **ODER** | 2x täglich (ohne Pause) |
| **Häufigkeit leichter Müdigkeit**  **→ Besserung durch kurze Ruhepausen** | **2 Tage im Monat** |  | **7 Tage im Monat** |
|  |  |  |  |
| Welches Medikament würden Sie wählen? (Kreuzen sie eines an) | Medikament A |  | Medikament B |

|  | **Medikament A** |  | **Medikament B** |
| --- | --- | --- | --- |
| Darreichung | Tablette |  | Tablette |
| Häufigkeit der Einnahme | 1x täglich (21/7 Schema) | **ODER** | 2x täglich (ohne Pause) |
| **Häufigkeit mittlerer oder starker Müdigkeit**  **→ keine Besserung durch Ruhepausen, Einschränkung im Alltag** | **7 Tage im Monat** |  | **2 Tage im Monat** |
|  |  |  |  |
| Welches Medikament würden Sie wählen? (Kreuzen sie eines an) | Medikament A |  | Medikament B |

|  | **Medikament A** |  | **Medikament B** |
| --- | --- | --- | --- |
| Darreichung | Tablette |  | Tablette |
| Häufigkeit der Einnahme | 1x täglich (21/7 Schema) | **ODER** | 2x täglich (ohne Pause) |
| **Häufigkeit mittlerer oder starker Müdigkeit**  **→ keine Besserung durch Ruhepausen, Einschränkung im Alltag** | **2 Tage im Monat** |  | **7 Tage im Monat** |
|  |  |  |  |
| Welches Medikament würden Sie wählen? (Kreuzen sie eines an) | Medikament A |  | Medikament B |

|  | **Medikament A** |  | **Medikament B** |
| --- | --- | --- | --- |
| Darreichung | Tablette |  | Tablette |
| Häufigkeit der Einnahme | 1x täglich (21/7 Schema) | **ODER** | 2x täglich (ohne Pause) |
| **Häufigkeit sehr starker Müdigkeit**  **→ keine Besserung durch Ruhepausen, Einschränkung in der Selbstversorgung** | **7 Tage im Monat** |  | **2 Tage im Monat** |
|  |  |  |  |
| Welches Medikament würden Sie wählen? (Kreuzen sie eines an) | Medikament A |  | Medikament B |

|  | **Medikament A** |  | **Medikament B** |
| --- | --- | --- | --- |
| Darreichung | Tablette |  | Tablette |
| Häufigkeit der Einnahme | 1x täglich (21/7 Schema) | **ODER** | 2x täglich (ohne Pause) |
| **Häufigkeit sehr starker Müdigkeit**  **→ keine Besserung durch Ruhepausen, Einschränkung in der Selbstversorgung** | **2 Tage im Monat** |  | **7 Tage im Monat** |
|  |  |  |  |
| Welches Medikament würden Sie wählen? (Kreuzen sie eines an) | Medikament A |  | Medikament B |
