# Supplementary material for: What are the needs in oral antitumor therapy? An analysis of patients’ and practitioners’ preferences
Source: Front Oncol. 2024 Jun 27;14:1388087. doi: 10.3389/fonc.2024.1388087 (PMC11236681; doi:10.3389/fonc.2024.1388087)
Supplement: Supplementary File 2 — Questionnaire for practitioners (in German). [file DataSheet_2.docx]

Sehr geehrte Kollegen und Kolleginnen, Mitarbeiter und Mitarbeiterinnen,

Seit einiger Zeit erhalten vor allem Patienten und Patientinnen zur Erstlinienbehandlung Ihres fortgeschrittenen Mammakarzinoms eine endokrin-basierte Therapie (z.B. Letrozol mit Ibrance®). Zur Verbesserung der Patientenbegleitung und des Zeitmanagements auf Seiten der Behandler in unserer Onkologischen Tagesklinik möchten wir Sie um Ihre Mithilfe bitten. Deshalb befragen wir Sie zu den Themen medizinische Untersuchungen, Aufklärungsgespräche von Patienten und zum Management vor der ersten Medikamenteneinnahme sowie im weiteren Verlauf. Nehmen Sie sich bitte kurz Zeit und beantworten Sie uns ein paar Fragen.

Bitte geben Sie den ausgefüllten Fragebogen in die dafür vorgesehenen Kästen im Stationsstützpunkt der Onkologischen Tagesklinik.

Für Rückfragen, Wünsche oder Anregungen stehen wir gerne zu Ihrer Verfügung.

Vielen Dank für Ihre Unterstützung

Prof. Dr. med. Nadia Harbeck, PD Dr. med. Rachel Würstlein und Team

| 1. **Angaben zur Person** | | | | | | | | | | | |
| --- | --- | --- | --- | --- | --- | --- | --- | --- | --- | --- | --- |
| 1. **Geschlecht:** männlich weiblich | | | | | | 1. **Alter:** ____________ | | | | | |
| 1. **Welche Tätigkeit üben Sie aktuell aus?** | | | | | | | | | | | |
| Arzt/Ärztin | | | Arzthelfer(in) | | | | | | | Breast Care Nurse | |
| Gesundheits- und Krankenpfleger(in) | | | | |  | | | | | | |
| Onkologische Fachgesundheits- und Krankenpfleger(in) | | | | | | | | | | | |
| Sonstige (bitte benennen): _________________________ | | | | | | | | | | | |
| 1. **In welcher Abteilung sind Sie beschäftigt?** | | | | | | | | | | | |
| Brustzentrum | | Onkologische Tagesklinik | | | | | |  | | | |
| Sonstige (bitte benennen): _________________________ | | | | | | | | | | | |
| 1. **Wie viele Jahre Berufserfahrung haben Sie in der Onkologie?** | | | | | | | | | | | |
| < 5 Jahre | | 5-10 Jahre | | | | | | | > 10 Jahre | | |
| 1. **Angaben zu Therapiepräferenzen** | | | | | | | | | | | |
| 1. **Welche der aufgeführten Darreichungsformen fallen Ihrer Meinung nach den Patienten(innen) unter onkologischer Therapie am leichtesten?** | | | | | | | | | | | |
|  | Trifft nicht zu | | | Trifft eher nicht zu | | | Trifft eher zu | | | | Trifft vollkommen zu |
| intravenös (i.v.) |  | | |  | | |  | | | |  |
| intramuskulär (i.m.) |  | | |  | | |  | | | |  |
| subkutan (s.c.) |  | | |  | | |  | | | |  |
| orale Therapie |  | | |  | | |  | | | |  |
| 1. **Welche Therapieintervalle bevorzugen Patienten(innen) unter onkologischer Therapie Ihrer Meinung nach?** | | | | | | | | | | | |
|  | Trifft nicht zu | | | Trifft eher nicht zu | | | Trifft eher zu | | | | Trifft vollkommen zu |
| wöchentlich |  | | |  | | |  | | | |  |
| 3-wöchentlich |  | | |  | | |  | | | |  |
| 4-wöchentlich |  | | |  | | |  | | | |  |
| vierteljährlich |  | | |  | | |  | | | |  |
| 1. **Welche der aufgeführten Darreichungsformen bevorzugen Sie in der klinischen Routine?** | | | | | | | | | | | |
|  | Trifft nicht zu | | | Trifft eher nicht zu | | | Trifft eher zu | | | | Trifft vollkommen zu |
| intravenös (i.v.) |  | | |  | | |  | | | |  |
| intramuskulär (i.m.) |  | | |  | | |  | | | |  |
| subkutan (s.c.) |  | | |  | | |  | | | |  |
| orale Therapie |  | | |  | | |  | | | |  |
| 1. **Welche Therapieintervalle bevorzugen Sie in der klinischen Routine?** | | | | | | | | | | | |
|  | Trifft nicht zu | | | Trifft eher nicht zu | | | Trifft eher zu | | | | Trifft vollkommen zu |
| wöchentlich |  | | |  | | |  | | | |  |
| 3-wöchentlich |  | | |  | | |  | | | |  |
| 4-wöchentlich |  | | |  | | |  | | | |  |
| vierteljährlich |  | | |  | | |  | | | |  |

| 1. **Welche der aufgeführten Schemata zur oralen Tumortherapie bevorzugen Ihrer Meinung nach Patienten(innen)?** | | | | |
| --- | --- | --- | --- | --- |
|  | Trifft nicht zu | Trifft eher nicht zu | Trifft eher zu | Trifft vollkommen zu |
| Kontinuierliche Tabletteneinnahme |  |  |  |  |
| 21/7 Schema: 21 Tage Einnahme/7 Tage Pause |  |  |  |  |
| 1. **Welche der aufgeführten Schemata zur oralen Tumortherapie bevorzugen Sie in der klinischen Routine?** | | | | |
|  | Trifft nicht zu | Trifft eher nicht zu | Trifft eher zu | Trifft vollkommen zu |
| Kontinuierliche Tabletteneinnahme |  |  |  |  |
| 21/7 Schema: 21 Tage Einnahme/7 Tage Pause |  |  |  |  |
| 1. **Empfinden Sie das eHealth basierte Nebenwirkungsmanagement (z.B. mit Cankado) als Erleichterung in der klinischen Routine?** | | | | |
|  | Trifft nicht zu | Trifft eher nicht zu | Trifft eher zu | Trifft vollkommen zu |
|  |  |  |  |  |

In diesem Fragenabschnitt möchten wir den medizinischen und pflegerischen Aufwand für Patienten(innen) analysieren, die sich zum ersten Mal mit der **fortgeschrittenen (metastasierten) Erkrankung** in Ihrer Abteilung vorstellen.

1. Wie erfolgt die Kontaktaufnahme vor Beginn der Therapie zwischen Patienten und Personal? Bitte kreuzen Sie an!

|  | **JA** | **NEIN** | **k.A.** |
| --- | --- | --- | --- |
| Patient(innen) kontaktieren uns telefonisch |  |  |  |
| Patient(innen) kontaktieren uns per E-Mail |  |  |  |
| Patient(innen) kommen persönlich zur Terminvereinbarung |  |  |  |
| Andere Kontaktaufnahme (bitte benennen): |  |  |  |

1. Wie oft stellen sich Patienten(innen) vor Therapiebeginn in Ihrer Abteilung vor?

| seltener | 1x | 1-2x | 2x | 2-3x | 3x | öfter |
| --- | --- | --- | --- | --- | --- | --- |
|  |  |  |  |  |  |  |

1. Bitte beantworten Sie folgende Aussagen

|  | Trifft nicht zu | Trifft eher nicht zu | Trifft eher zu | Trifft vollkommen zu |
| --- | --- | --- | --- | --- |
| Alle erforderlichen Untersuchungsergebnisse liegen zum Zeitpunkt der Erstvorstellung vor |  |  |  |  |
| Zusätzlich notwendige Untersuchungen werden von Ihnen organisiert |  |  |  |  |
| Untersuchungsergebnisse werden von Ihnen angefordert |  |  |  |  |
| Die Vorbereitung und Durchführung von Laboruntersuchungen erfolgt durch den Arzt |  |  |  |  |
| Die Vorbereitung und Durchführung von Laboruntersuchungen erfolgt durch das Pflegepersonal |  |  |  |  |
| Die Kooperation mit niedergelassenen Ärzten erfolgt reibungslos |  |  |  |  |

1. Wenn Sie in die folgenden Tätigkeiten involviert sind, dann geben Sie bitte den Zeitaufwand vor Beginn der Therapie an. Ansonsten lassen Sie die Zeile bitte frei.

|  | Zeit (in Minuten) |
| --- | --- |
| Telefonat/Gespräch mit Patient(innen) zur Terminvereinbarung |  |
| Prüfung von Untersuchungsergebnissen auf Vollständigkeit, Anlegen einer Patientenakte etc. |  |
| Anforderung von Untersuchungsergebnissen aus externen Praxen |  |
| Einscannen externer Befunde ins klinikinterne KAS System |  |
| Vorbereitung und Durchführung von Laboruntersuchungen |  |
| Vorbereitung bzw. Veranlassung zusätzlicher Untersuchungen |  |
| Vorbereitung der Besprechung im interdisziplinären Tumorboard |  |
| Vorbereitung aller Unterlagen zur Therapieaufklärung (Aufklärung vorbereiten/drucken, Brief erstellen etc.) |  |
| Aufklärungsgespräch über die anstehende Therapie inkl. aller empfohlenen Substanzen |  |
| Erklärung des Therapieablaufs (anstehende Termine, Aushändigen des Nachsorgekalender etc.) |  |
| Terminierung der nächsten Besuche |  |
| Verlaufstabelle und -dokumentation im KAS |  |

1. Offene Frage

| Welche zusätzlichen Vorbereitungen oder Untersuchungen (inkl. Blutabnahmen etc.) müssen vor einer Therapieaufklärung speziell von Ihnen getroffen werden?  Wieviel Zeit nimmt die Vorbereitung und die Durchführung in Anspruch? |  |
| --- | --- |

Im nächsten Abschnitt möchten wir den medizinischen und pflegerischen Aufwand für Patienten(innen) analysieren, die sich **innerhalb der ersten** **3 Monate nach Therapiebeginn** in Ihrer Abteilung vorstellen.

1. Wie erfolgt die Kontaktaufnahme zwischen Patienten und Personal? Bitte kreuzen Sie an!

|  | **JA** | **NEIN** | **k.A.** |
| --- | --- | --- | --- |
| Patient(innen) kontaktieren uns telefonisch |  |  |  |
| Patient(innen) kontaktieren uns per E-Mail |  |  |  |
| Patient(innen) kommen persönlich zur Terminvereinbarung |  |  |  |
| Andere Kontaktaufnahme (bitte benennen): |  |  |  |

1. Wie oft stellen sich Patienten(innen) in den ersten 3 Monaten nach Therapiebeginn in Ihrer Abteilung vor?

| seltener | 1x | 1-2x | 2x | 2-3x | 3x | öfter |
| --- | --- | --- | --- | --- | --- | --- |
|  |  |  |  |  |  |  |

1. Bitte beantworten Sie folgende Aussagen

|  | Trifft nicht zu | | Trifft eher nicht zu | Trifft eher zu | Trifft vollkommen zu |
| --- | --- | --- | --- | --- | --- |
| In den ersten beiden Zyklen kommen die Patienten(innen) alle 2 Wochen zur Blutbildkontrolle |  |  | |  |  |
| Ab Zyklus 3 kommen die Patienten(innen) 1x im Monat zur Blutbildkontrolle |  |  | |  |  |
| Die Blutbildkontrollen erfolgen in den ersten 3 Monaten in der Onkologischen Tagesklinik |  |  | |  |  |
| Die Vorbereitung und Durchführung von Laboruntersuchungen erfolgt durch Sie |  |  | |  |  |
| Die Blutbildkontrollen erfolgen in den ersten 3 Monaten beim Hausarzt |  |  | |  |  |
| Die Ergebnisse der Blutbild-kontrollen vom Hausarzt sind vollständig |  |  | |  |  |
| Zusätzliche oder fehlende Untersuchungsergebnisse werden von Ihnen angefordert |  |  | |  |  |
| Die Kooperation mit niedergelassenen Ärzten erfolgt reibungslos |  |  | |  |  |

1. Aufgrund welcher Komplikationen melden sich Patienten(innen) in den ersten 3 Behandlungsmonaten nach Beginn der Therapie außerhalb der routinemäßigen Termine in Ihrer Abteilung?

| Schmerzen | Blutbild-veränderungen | Infektionen | Durchfall | Fieber | Angstzustände, Psyche | Sonstiges |
| --- | --- | --- | --- | --- | --- | --- |
|  |  |  |  |  |  |  |

Sonstige (bitte benennen): _____________________________

1. Wenn Sie in die folgenden Tätigkeiten involviert sind, dann geben Sie bitte den Zeitaufwand in den ersten 3 Monaten der endokrin-basierten Therapie an. Ansonsten lassen Sie die Zeile bitte frei.

|  | Zeitaufwand (in Minuten) |
| --- | --- |
| Telefonat mit Patient(innen) zur Klärung des unerwünschten Ereignisses und ggf. Neuterminierung |  |
| Regelmäßige Gespräche zu Fragen, Nebenwirkungen etc. zur aktuellen Therapie |  |
| Prüfung von Untersuchungsergebnissen auf Vollständigkeit |  |
| Anforderung von Untersuchungsergebnissen aus externen Praxen |  |
| Vorbereitung und Durchführung von Laboruntersuchungen |  |
| Vorbereitung bzw. Veranlassung zusätzlicher Untersuchungen |  |
| Rücksprache mit Oberärzten zur Klärung des Vorgehens von unerwünschten Ereignissen |  |
| Terminierung der nächsten Routine-Besuche |  |
| Verlaufstabelle und -dokumentation in der Akte/im KAS |  |

1. Offene Frage

| Welche zusätzlichen Aufgaben müssen von Ihnen speziell durchgeführt werden?  Wieviel Zeit nimmt das Vorgehen in Anspruch? |  |
| --- | --- |

In diesem Abschnitt möchten wir den medizinischen und pflegerischen Aufwand für Patienten(innen) analysieren, die **mehr als 3 Monate die endokrin-basierte Therapie** erhalten und sich regelmäßig in Ihrer Abteilung vorstellen.

1. Wie erfolgt die Kontaktaufnahme zwischen Patienten und Personal?

|  | **JA** | **NEIN** | **Keine Angabe** |
| --- | --- | --- | --- |
| Patient(innen) kontaktieren uns telefonisch |  |  |  |
| Patient(innen) kontaktieren uns per E-Mail |  |  |  |
| Patient(innen) kommen persönlich zur Terminvereinbarung |  |  |  |
| Andere Kontaktaufnahme (bitte benennen): |  |  |  |

1. Bitte beantworten Sie folgende Aussagen

|  | Trifft nicht zu | | Trifft eher nicht zu | Trifft eher zu | Trifft vollkommen zu |
| --- | --- | --- | --- | --- | --- |
| Die Patienten(innen) kommen 1x im Monat zur Blutbildkontrolle |  |  | |  |  |
| Die Patienten(innen) kommen alle 3 Monate zur Blutbildkontrolle |  |  | |  |  |
| Die Patienten(innen) kommen seltener als alle 3 Monate zur Blutbildkontrolle |  |  | |  |  |
| Die Blutbildkontrollen erfolgen in der Onkologischen Tagesklinik |  |  | |  |  |
| Die Blutbildkontrollen erfolgen beim Hausarzt |  |  | |  |  |
| Die Vorbereitung und Durchführung von Laboruntersuchungen erfolgt durch Sie |  |  | |  |  |
| Die Ergebnisse der Blutbildkontrollen vom Hausarzt sind vollständig |  |  | |  |  |
| Zusätzliche oder fehlende Untersuchungsergebnisse werden von Ihnen angefordert |  |  | |  |  |
| Die Kooperation mit niedergelassenen Ärzten erfolgt reibungslos |  |  | |  |  |

1. Aufgrund welcher Komplikationen stellen sich Patienten(innen) im weiteren Therapieverlauf außerhalb der routinemäßigen Termine in Ihrer Abteilung vor?

| Schmerzen | Blutbild-veränderungen | Infektionen | Durchfall | Fieber | Angstzustände, Psyche | Sonstiges |
| --- | --- | --- | --- | --- | --- | --- |
|  |  |  |  |  |  |  |

Sonstige (bitte benennen): _____________________________

1. Wenn Sie in die folgenden Tätigkeiten involviert sind, dann geben Sie bitte den Zeitaufwand im weiteren Verlauf (˃ 3 Monate) für Patientinnen mit endokrin-basierten Therapie an. Ansonsten lassen Sie die Zeile bitte frei.

|  | Zeitaufwand (in Minuten) |
| --- | --- |
| Telefonat mit Patient(innen) zur Klärung des unerwünschten Ereignisses und ggf. Neuterminierung |  |
| Regelmäßige Gespräche zu Fragen, Nebenwirkungen etc. zur aktuellen Therapie |  |
| Prüfung von Untersuchungsergebnissen auf Vollständigkeit |  |
| Anforderung von Untersuchungsergebnissen aus externen Praxen |  |
| Vorbereitung und Durchführung von Laboruntersuchungen |  |
| Vorbereitung bzw. Veranlassung zusätzlicher Untersuchungen |  |
| Rücksprache mit Oberärzten zur Klärung des Vorgehens von unerwünschten Ereignissen |  |
| Terminierung der nächsten Routine-Besuche |  |
| Verlaufstabelle und -dokumentation in der Akte/im KAS |  |

1. Offene Frage

| Welche zusätzlichen Aufgaben müssen von Ihnen speziell durchgeführt werden?  Wieviel Zeit nimmt das Vorgehen in Anspruch? |  |
| --- | --- |
