# Supplementary material for: What are the needs in oral antitumor therapy? An analysis of patients’ and practitioners’ preferences
Source: Front Oncol. 2024 Jun 27;14:1388087. doi: 10.3389/fonc.2024.1388087 (PMC11236681; doi:10.3389/fonc.2024.1388087)
Supplement: Supplementary File 3 — Questionnaire for patients (translated to English for submission). [file DataSheet_3.docx]

Dear patient,

You are currently receiving anti-tumor therapy in our oncology day clinic to treat your advanced breast cancer. We would like to optimize your medical care and the amount of time you spend visiting us during this therapy. We would therefore like to ask for your help by completing this questionnaire. In it, we ask you demographic questions about yourself, your disease and your current therapy. We would then like to know how this therapy can be integrated into your everyday life and which therapies you would prefer.

The questionnaires are marked in different colors so that your answers can be assigned to your current therapy (e.g. chemotherapy, anti-hormonal therapy or combination therapy) and evaluated accordingly. As only a small number of patients are surveyed (approx. 60 in total), the risk of re-identification is increased. Your answers will of course be treated confidentially and processed without your personal data. Therefore, please complete this questionnaire and place it in the boxes provided for this purpose in the ward base of the oncology day clinic.

The pharmaceutical company Lilly Deutschland GmbH is supporting this project financially in order to cover any additional costs incurred. There is no financial compensation for you as a participant for answering the questionnaires. After the evaluation, only complete data sets, without personal identifying characteristics, will be transmitted to the sponsor.

By submitting the questionnaire, you give your consent to our project. Revocation and deletion of the data collected is no longer possible thereafter.

Dr. Rachel Wuerstlein and her team will be happy to answer any questions, suggestions and requests you may have.

Thank you for your support

Prof. Dr. med. Nadia Harbeck, PD Dr. med. Rachel Wuerstlein and team

Please answer each of the questions by selecting the answer option that best applies to you or by entering the corresponding answer.

| 1. **Personal details** | | | | | | | | | | | |
| --- | --- | --- | --- | --- | --- | --- | --- | --- | --- | --- | --- |
| 1. **Gender:** male female | | | | | | 1. **Year of birth:** ____________ | | | | | |
| 1. **What is your highest educational qualification?** | | | | | | | | | | | |
| Secondary school certificate/elementary school | | | | Secondary school certificate/intermediate school leaving certificate | | | | | A-levels | | |
| Bachelor's degree | Master/Diploma | | | | | | Promotion | | | | Habilitation |
| 1. **To what extent are you employed?** | | | | | | | | | | | |
| Full-time | | | Part-time | | | | | Student/Trainee | | | |
| Pensioner | | | voluntary work | | | | | unoccupied | | | |
| 1. **What is your family situation like?** | | | | | | | | | | | |
| single | | | Divorced | | | | | Civil partnership annulled | | | |
| married | | | widowed | | | | | Life partner deceased | | | |
| Living separately | | | Life partnership | | | | |  | | | |
| 1. **Do you have children?** | | | | | | | | | | | |
| Yes No | | | If yes: How many? _______________ | | | | | | | | |
| 1. **Details of your illness** | | | | | | | | | | | |
| 1. **When was your illness first diagnosed (year)?** _________________ | | | | | | | | | | | |
| 1. **How long has it been known that your tumor has spread (year)?** _________________ | | | | | | | | | | | |
| 1. **Which organs are affected by the metastases?** | | | | | | | | | | | |
| Bones Lungs/pleura Liver Skin Brain Other: __________________ | | | | | | | | | | | |
| 1. **Details of your therapy** | | | | | | | | | | | |
| 1. **Are you receiving therapy to maintain bone stability (e.g. Bondronat, X-GEVA)?** | | | | | | | | | | | |
| Yes No | | | If yes: which one? _______________________ | | | | | | | | |
| 1. **Which tumor therapy are you currently receiving?** | | | | | | | | | | | |
| chemotherapy (e.g. paclitaxel, Avastin)  anti-hormonal therapy (e.g. Tamoxifen®, Letrozole®, Faslodex®) | | | | | | | | | | | |
| a combination therapy (e.g. Ibrance® or Verzenios® with Letrozole® or Faslodex®) | | | | | | | | | | | |
| 1. **Are you receiving therapy to suppress ovarian function (e.g. with Zoladex®)?** | | | | | | | | | | | |
| Yes No | | | If yes: which one? _______________________ | | | | | | | | |
|  | | |  | | | | | | | | |
|  | | |  | | | | | | | | |
| 1. **Wishes for your therapy** | | | | | | | | | | | |
| 1. **If you had the choice, which form of medication administration would you prefer?** | | | | | | | | | | | |
|  | | Strongly disagree | | | Partially disagree | | | Partially agree | | Strongly agree | |
| Tablet | |  | | |  | | |  | |  | |
| Injection into fatty tissue | |  | | |  | | |  | |  | |
| Injection into the muscle | |  | | |  | | |  | |  | |
| Infusion (intravenous) | |  | | |  | | |  | |  | |
| 1. **How many tablets do you take in total in one day?** | | | | | | | | | | | |
| 1 tablet 2 tablets 3 tablets 4 tablets 5 tablets | | | | | | | | | | | |
| 6 tablets 7 tablets 8 tablets 9 tablets 10 tablets | | | | | | | | | | | |
| more than 10 tablets | | | | | | | | | | | |
| 1. **If you could reduce the side effects of tumor therapy by taking more tablets, how many additional tablets would you take?** | | | | | | | | | | | |
| 1 tablet 2 tablets 3 tablets 4 tablets 5 tablets | | | | | | | | | | | |
| more than 5 tablets | | | | | | | | | | | |
| 1. **Do you use the following aids to remind you to take your tablets?** | | | | | | | | | | | |
|  | | Strongly disagree | | | Partially disagree | | | Partially agree | | Strongly agree | |
| Diary | |  | | |  | | |  | |  | |
| Calendar | |  | | |  | | |  | |  | |
| App on the smartphone | |  | | |  | | |  | |  | |
| Other: ____________________ | |  | | |  | | |  | |  | |
| 1. **If you were allowed to determine your own visits to the oncology day clinic, at what intervals would you prefer to come?** | | | | | | | | | | | |
|  | | Strongly disagree | | | Partially disagree | | | Partially agree | | Strongly agree | |
| weekly | |  | | |  | | |  | |  | |
| 3-weekly | |  | | |  | | |  | |  | |
| monthly | |  | | |  | | |  | |  | |
| every 3 months | |  | | |  | | |  | |  | |
| 1. **Can you integrate your current therapy well into the following life situations?** | | | | | | | | | | | |
|  | | Strongly disagree | | | Partially disagree | | | Partially agree | | Strongly agree | |
| in everyday life | |  | | |  | | |  | |  | |
| in work | |  | | |  | | |  | |  | |
| during leisure time | |  | | |  | | |  | |  | |
| for vacation trips | |  | | |  | | |  | |  | |
| 1. **I would like to have a continuous contact person for the therapy time in our oncology day clinic.** | | | | | | | | | | | |
|  | | Strongly disagree | | | Partially disagree | | | Partially agree | | Strongly agree | |
|  | |  | | |  | | |  | |  | |

| 1. **This contact person should belong to the following professional group:** | | | | |
| --- | --- | --- | --- | --- |
|  | Strongly disagree | Partially disagree | Partially agree | Strongly agree |
| physician |  |  |  |  |
| Specially trained nursing staff in oncology |  |  |  |  |
| 1. **Have you ever consulted or read up on the following instances during the current therapy due to general questions?** | | | | |
|  | Strongly disagree | Partially disagree | Partially agree | Strongly agree |
| Internet |  |  |  |  |
| Patient avocacy group |  |  |  |  |
| Group chats |  |  |  |  |
| other patients |  |  |  |  |
| Emergency department |  |  |  |  |
| Outpatient oncology unit |  |  |  |  |
| 1. **Have you ever contacted or read up on the following instances due to side effects during your current therapy?** | | | | |
|  | Strongly disagree | Partially disagree | Partially agree | Strongly agree |
| Internet |  |  |  |  |
| Patient avocacy group |  |  |  |  |
| Group chats |  |  |  |  |
| other patients |  |  |  |  |
| Emergency department |  |  |  |  |
| Outpatient oncology unit |  |  |  |  |

Dear patient,

On the following pages, 2 drugs for tumor therapy in tablet form for the treatment of metastatic breast cancer are presented several times. These are not actual drugs, but fictitious drugs that have been invented for research purposes. We would like to ask you to compare the drugs and indicate which drug you prefer if they differ **only** in the characteristics indicated. You should assume that **both drugs have the same efficacy** and are otherwise similar in all other respects except for the stated characteristics. Each of your assessments is important to us. Please do not leave any out, even if a comparison may be difficult.

Which of the two drugs would you prefer?

**Take medication A** once a day at the same time every day. Take it for 21 days followed by a 7-day break (21/7 schedule) and so on.

**Take medication B** twice a day, i.e. in the morning and evening at the same time. Take the medication continuously without a break.

1. General preference

|  | **Drug A** |  | **Drug B** |
| --- | --- | --- | --- |
| Presentation | Tablet |  | Tablet |
| Frequency of use | 1x daily (21/7 scheme) | **OR** | 2x daily (without break) |
|  |  |  |  |
| Which medication would you choose? (Check one) | Drug A |  | Drug B |

1. Side effect: Neutropenia (low white blood cell count)

|  | **Drug A** |  | **Drug B** |
| --- | --- | --- | --- |
| Presentation | Tablet |  | Tablet |
| Frequency of use | 1x daily (21/7 scheme) | **OR** | 2x daily (without break) |
| **Frequency of low white blood cell counts and increased risk of infection** | **7 days a month** |  | **2 days per month** |
|  |  |  |  |
| Which medication would you choose? (Check one) | Drug A |  | Drug B |

|  | **Drug A** |  | **Drug B** |
| --- | --- | --- | --- |
| Presentation | Tablet |  | Tablet |
| Frequency of use | 1x daily (21/7 scheme) | **OR** | 2x daily (without break) |
| **Frequency of low white blood cell counts and increased risk of infection** | **2 days per month** |  | **7 days a month** |
|  |  |  |  |
| Which medication would you choose? (Check one) | Drug A |  | Drug B |

1. Side effect: discomfort in the hands and/or feet

|  | **Drug A** |  | **Drug B** |
| --- | --- | --- | --- |
| Presentation | Tablet |  | Tablet |
| Frequency of use | 1x daily (21/7 scheme) | **OR** | 2x daily (without break) |
| **Frequency of slight discomfort in fingertips and toes** | **7 days a month** |  | **2 days per month** |
|  |  |  |  |
| Which medication would you choose? (Check one) | Drug A |  | Drug B |

|  | **Drug A** |  | **Drug B** |
| --- | --- | --- | --- |
| Presentation | Tablet |  | Tablet |
| Frequency of use | 1x daily (21/7 scheme) | **OR** | 2x daily (without break) |
| **Frequency of slight discomfort in fingertips and toes** | **2 days per month** |  | **7 days a month** |
|  |  |  |  |
| Which medication would you choose? (Check one) | Drug A |  | Drug B |

|  | **Drug A** |  | **Drug B** |
| --- | --- | --- | --- |
| Presentation | Tablet |  | Tablet |
| Frequency of use | 1x daily (21/7 scheme) | **OR** | 2x daily (without break) |
| **Frequency of sensory disturbances, loss of sensation (limited function in everyday life)** | **7 days a month** |  | **2 days per month** |
|  |  |  |  |
| Which medication would you choose? (Check one) | Drug A |  | Drug B |

|  | **Drug A** |  | **Drug B** |
| --- | --- | --- | --- |
| Presentation | Tablet |  | Tablet |
| Frequency of use | 1x daily (21/7 scheme) | **OR** | 2x daily (without break) |
| **Frequency of sensory disturbances, loss of sensation (limited function in everyday life)** | **2 days per month** |  | **7 days a month** |
|  |  |  |  |
| Which medication would you choose? (Check one) | Drug A |  | Drug B |

|  | **Drug A** |  | **Drug B** |
| --- | --- | --- | --- |
| Presentation | Tablet |  | Tablet |
| Frequency of use | 1x daily (21/7 scheme) | **OR** | 2x daily (without break) |
| **Frequency of severe discomfort**  **→ Restriction of self-sufficiency** | **7 days a month** |  | **2 days per month** |
|  |  |  |  |
| Which medication would you choose? (Check one) | Drug A |  | Drug B |

|  | **Drug A** |  | **Drug B** |
| --- | --- | --- | --- |
| Presentation | Tablet |  | Tablet |
| Frequency of use | 1x daily (21/7 scheme) | **OR** | 2x daily (without break) |
| **Frequency of severe discomfort**  **→ Restriction of self-sufficiency** | **2 days per month** |  | **7 days a month** |
|  |  |  |  |
| Which medication would you choose? (Check one) | Drug A |  | Drug B |

1. Side effect: Diarrhea

|  | **Drug A** |  | **Drug B** |
| --- | --- | --- | --- |
| Presentation | Tablet |  | Tablet |
| Frequency of use | 1x daily (21/7 schedule) | **OR** | 2x daily (without break) |
| **Frequency of increased bowel movements (up to once a day)** | **7 days a month** |  | **2 days per month** |
|  |  |  |  |
| Which medication would you choose? (Check one) | Drug A |  | Drug B |

|  | **Drug A** |  | **Drug B** |
| --- | --- | --- | --- |
| Presentation | Tablet |  | Tablet |
| Frequency of use | 1x daily (21/7 schedule) | **OR** | 2x daily (without break) |
| **Frequency of increased bowel movements (up to once a day)** | **2 days per month** |  | **7 days a month** |
|  |  |  |  |
| Which medication would you choose? (Check one) | Drug A |  | Drug B |

|  | **Drug A** |  | **Drug B** |
| --- | --- | --- | --- |
| Presentation | Tablet |  | Tablet |
| Frequency of use | 1x daily (21/7 schedule) | **OR** | 2x daily (without break) |
| **Frequency of increased bowel movements (2-5x daily)** | **7 days a month** |  | **2 days per month** |
|  |  |  |  |
| Which medication would you choose? (Check one) | Drug A |  | Drug B |

|  | **Drug A** |  | **Drug B** |
| --- | --- | --- | --- |
| Presentation | Tablet |  | Tablet |
| Frequency of use | 1x daily (21/7 schedule) | **OR** | 2x daily (without break) |
| **Frequency of increased bowel movements (2-5x daily)** | **2 days per month** |  | **7 days a month** |
|  |  |  |  |
| Which medication would you choose? (Check one) | Drug A |  | Drug B |

|  | **Drug A** |  | **Drug B** |
| --- | --- | --- | --- |
| Presentation | Tablet |  | Tablet |
| Frequency of use | 1x daily (21/7 schedule) | **OR** | 2x daily (without break) |
| **Frequency of increased bowel movements (more than 6 times a day)** | **7 days a month** |  | **2 days per month** |
|  |  |  |  |
| Which medication would you choose? (Check one) | Drug A |  | Drug B |

|  | **Drug A** |  | **Drug B** |
| --- | --- | --- | --- |
| Presentation | Tablet |  | Tablet |
| Frequency of use | 1x daily (21/7 schedule) | **OR** | 2x daily (without break) |
| **Frequency of increased bowel movements (more than 6 times a day)** | **2 days per month** |  | **7 days a month** |
|  |  |  |  |
| Which medication would you choose? (Check one) | Drug A |  | Drug B |

1. Side effect: fatigue/tiredness

|  | **Drug A** |  | **Drug B** |
| --- | --- | --- | --- |
| Presentation | Tablet |  | Tablet |
| Frequency of use | 1x daily (21/7 scheme) | **OR** | 2x daily (without break) |
| **Frequency of mild fatigue**  **→ Improvement through short rest breaks** | **7 days a month** |  | **2 days per month** |
|  |  |  |  |
| Which medication would you choose? (Check one) | Drug A |  | Drug B |

|  | **Drug A** |  | **Drug B** |
| --- | --- | --- | --- |
| Presentation | Tablet |  | Tablet |
| Frequency of use | 1x daily (21/7 scheme) | **OR** | 2x daily (without break) |
| **Frequency of mild fatigue**  **→ Improvement through short rest breaks** | **2 days per month** |  | **7 days a month** |
|  |  |  |  |
| Which medication would you choose? (Check one) | Drug A |  | Drug B |

|  | **Drug A** |  | **Drug B** |
| --- | --- | --- | --- |
| Presentation | Tablet |  | Tablet |
| Frequency of use | 1x daily (21/7 scheme) | **OR** | 2x daily (without break) |
| **Frequency of moderate or severe fatigue**  **→ No improvement due to rest breaks, restriction in everyday life** | **7 days a month** |  | **2 days per month** |
|  |  |  |  |
| Which medication would you choose? (Check one) | Drug A |  | Drug B |

|  | **Drug A** |  | **Drug B** |
| --- | --- | --- | --- |
| Presentation | Tablet |  | Tablet |
| Frequency of use | 1x daily (21/7 scheme) | **OR** | 2x daily (without break) |
| **Frequency of moderate or severe fatigue**  **→ No improvement due to rest breaks, restriction in everyday life** | **2 days per month** |  | **7 days a month** |
|  |  |  |  |
| Which medication would you choose? (Check one) | Drug A |  | Drug B |

|  | **Drug A** |  | **Drug B** |
| --- | --- | --- | --- |
| Presentation | Tablet |  | Tablet |
| Frequency of use | 1x daily (21/7 scheme) | **OR** | 2x daily (without break) |
| **Frequency of very severe fatigue**  **→ No improvement through rest breaks, restriction in self-care** | **7 days a month** |  | **2 days per month** |
|  |  |  |  |
| Which medication would you choose? (Check one) | Drug A |  | Drug B |

|  | **Drug A** |  | **Drug B** |
| --- | --- | --- | --- |
| Presentation | Tablet |  | Tablet |
| Frequency of use | 1x daily (21/7 scheme) | **OR** | 2x daily (without break) |
| **Frequency of very severe fatigue**  **→ No improvement through rest breaks, restriction in self-care** | **2 days per month** |  | **7 days a month** |
|  |  |  |  |
| Which medication would you choose? (Check one) | Drug A |  | Drug B |
