# Supplementary material for: What are the needs in oral antitumor therapy? An analysis of patients’ and practitioners’ preferences
Source: Front Oncol. 2024 Jun 27;14:1388087. doi: 10.3389/fonc.2024.1388087 (PMC11236681; doi:10.3389/fonc.2024.1388087)
Supplement: Supplementary File 4 — Questionnaire for practitioners (translated to English for submission, shortened to the questions relevant for this publication). [file DataSheet_4.docx]

Dear colleagues and employees,

For some time now, patients have been frequently receiving endocrine-based therapy (e.g. Letrozole with Ibrance®) as first-line treatment for their advanced breast cancer. We would like to ask for your help to improve patient care and time management on the part of the practitioners in our oncology day clinic. We would therefore like to ask you about medical examinations, patient information discussions and management before the first dose of medication and during the further course of treatment. Please take a few moments to answer a few questions.

Please place the completed questionnaire in the boxes provided in the ward base of the oncology day clinic.

If you have any questions, requests or suggestions, please do not hesitate to contact us.

Thank you for your support

Prof. Dr. med. Nadia Harbeck, PD Dr. med. Rachel Wuerstlein and team

| 1. **Personal details** | | | | | | | | | | | |
| --- | --- | --- | --- | --- | --- | --- | --- | --- | --- | --- | --- |
| 1. **Gender:** male female | | | | | | 1. **Age:** ____________ | | | | | |
| 1. **What is your current job?** | | | | | | | | | | | |
| Doctor | | | Medical assistant | | | | | | | Breast Care Nurse | |
| Nurse | | | | |  | | | | | | |
| nurse specialized in oncology | | | | | | | | | | | |
| Other (please specify): _________________________ | | | | | | | | | | | |
| 1. **In which department are you employed?** | | | | | | | | | | | |
| Breast Center | | Oncology day clinic | | | | | |  | | | |
| Other (please specify): _________________________ | | | | | | | | | | | |
| 1. **How many years of professional experience do you have in oncology?** | | | | | | | | | | | |
| < 5 years | | 5-10 years | | | | | | | > 10 years | | |
| 1. **Information on treatment preferences** | | | | | | | | | | | |
| 1. **In your opinion, which of the listed dosage forms are easiest for patients undergoing oncological therapy?** | | | | | | | | | | | |
|  | Does not apply | | | Rather not applicable | | | Rather true | | | | Fully applicable |
| intravenous (i.v.) |  | | |  | | |  | | | |  |
| intramuscular (i.m.) |  | | |  | | |  | | | |  |
| subcutaneously (s.c.) |  | | |  | | |  | | | |  |
| oral therapy |  | | |  | | |  | | | |  |
| 1. **In your opinion, which therapy intervals do patients undergoing oncological therapy prefer?** | | | | | | | | | | | |
|  | Does not apply | | | Rather not applicable | | | Rather true | | | | Fully applicable |
| weekly |  | | |  | | |  | | | |  |
| 3-weekly |  | | |  | | |  | | | |  |
| 4-weekly |  | | |  | | |  | | | |  |
| quarterly |  | | |  | | |  | | | |  |
| 1. **Which of the listed dosage forms do you prefer in clinical routine?** | | | | | | | | | | | |
|  | Does not apply | | | Rather not applicable | | | Rather true | | | | Fully applicable |
| intravenous (i.v.) |  | | |  | | |  | | | |  |
| intramuscular (i.m.) |  | | |  | | |  | | | |  |
| subcutaneously (s.c.) |  | | |  | | |  | | | |  |
| oral therapy |  | | |  | | |  | | | |  |
| 1. **Which therapy intervals do you prefer in routine clinical practice?** | | | | | | | | | | | |
|  | Does not apply | | | Rather not applicable | | | Rather true | | | | Fully applicable |
| weekly |  | | |  | | |  | | | |  |
| 3-weekly |  | | |  | | |  | | | |  |
| 4-weekly |  | | |  | | |  | | | |  |
| quarterly |  | | |  | | |  | | | |  |

| 1. **In your opinion, which of the listed regimens for oral tumor therapy do patients prefer?** | | | | |
| --- | --- | --- | --- | --- |
|  | Does not apply | Rather not applicable | Rather true | Fully applicable |
| Continuous tablet intake |  |  |  |  |
| 21/7 scheme: 21 days intake/7 days break |  |  |  |  |
| 1. **Which of the listed regimens for oral tumor therapy do you prefer in routine clinical practice?** | | | | |
|  | Does not apply | Rather not applicable | Rather true | Fully applicable |
| Continuous tablet intake |  |  |  |  |
| 21/7 scheme: 21 days intake/7 days break |  |  |  |  |
| 1. **Do you feel that eHealth-based side effect management (e.g. with Cankado) makes clinical routine easier?** | | | | |
|  | Does not apply | Rather not applicable | Rather true | Fully applicable |
|  |  |  |  |  |
